# Gene Duplication in the Sugarcane Genome: A Case Study of Allele Interactions and Evolutionary Patterns in Two Genic Regions

**DOI:** 10.3389/fpls.2019.00553

**Published:** 2019-05-07

**Authors:** Danilo Augusto Sforça, Sonia Vautrin, Claudio Benicio Cardoso-Silva, Melina Cristina Mancini, María Victoria Romero-da Cruz, Guilherme da Silva Pereira, Mônica Conte, Arnaud Bellec, Nair Dahmer, Joelle Fourment, Nathalie Rodde, Marie-Anne Van Sluys, Renato Vicentini, Antônio Augusto Franco Garcia, Eliana Regina Forni-Martins, Monalisa Sampaio Carneiro, Hermann Paulo Hoffmann, Luciana Rossini Pinto, Marcos Guimarães de Andrade Landell, Michel Vincentz, Helene Berges, Anete Pereira de Souza

**Affiliations:** ^1^Universidade Estadual de Campinas (UNICAMP), Campinas, Brazil; ^2^Centre National de Ressources Genomiques Vegetales (CNRGV), Institut National de la Recherche Agronomique (INRA), Castanet Tolosan, France; ^3^Escola Superior de Agricultura Luiz de Queiroz (ESALQ), USP, Piracicaba, Brazil; ^4^Instituto de Biociências, Universidade de São Paulo (USP), São Paulo, Brazil; ^5^Centro de Ciências Agrárias, Universidade Federal de São Carlos (UFSCAR), Araras, Brazil; ^6^Centro de Cana, Instituto Agronômico de Campinas (IAC), Ribeirão Preto, Brazil

**Keywords:** chimerical gene, genetic mapping, homologs, physical mapping, polyploid, sugarcane

## Abstract

Sugarcane (*Saccharum* spp.) is highly polyploid and aneuploid. Modern cultivars are derived from hybridization between *S. officinarum* and *S. spontaneum*. This combination results in a genome exhibiting variable ploidy among different loci, a huge genome size (~10 Gb) and a high content of repetitive regions. An approach using genomic, transcriptomic, and genetic mapping can improve our knowledge of the behavior of genetics in sugarcane. The hypothetical *HP600* and Centromere Protein C (*CENP-C*) genes from sugarcane were used to elucidate the allelic expression and genomic and genetic behaviors of this complex polyploid. The physically linked side-by-side genes *HP600* and *CENP-C* were found in two different homeologous chromosome groups with ploidies of eight and ten. The first region (Region01) was a *Sorghum bicolor* ortholog region with all haplotypes of *HP600* and *CENP-C* expressed, but *HP600* exhibited an unbalanced haplotype expression. The second region (Region02) was a scrambled sugarcane sequence formed from different noncollinear genes containing partial duplications of *HP600* and *CENP-C* (paralogs). This duplication resulted in a non-expressed *HP600* pseudogene and a recombined fusion version of *CENP-C* and the orthologous gene Sobic.003G299500 with at least two chimeric gene haplotypes expressed. It was also determined that it occurred before *Saccharum* genus formation and after the separation of sorghum and sugarcane. A linkage map was constructed using markers from nonduplicated Region01 and for the duplication (Region01 and Region02). We compare the physical and linkage maps, demonstrating the possibility of mapping markers located in duplicated regions with markers in nonduplicated region. Our results contribute directly to the improvement of linkage mapping in complex polyploids and improve the integration of physical and genetic data for sugarcane breeding programs. Thus, we describe the complexity involved in sugarcane genetics and genomics and allelic dynamics, which can be useful for understanding complex polyploid genomes.

## Introduction

The *Saccharum* species are C4 grasses and present a high level of ploidy. *S. officinarum* L. is an octaploid (2n = 80) with *x* = 10 chromosomes, while *S. spontaneum* L. has *x* = 8 but presents great variations in the number of chromosomes, with main the cytotypes of 2n = 62, 80, 96, 112, or 128. Modern sugarcane cultivars originated from hybridization between these two species (Daniels and Roach, 1987; Paterson et al., 2013). The development of these cultivars involved the process of “nobilization” of the hybrid, with successive backcrosses using *S. officinarum* as the recurrent parent (D'Hont et al., 1998). The resulting hybrids are highly polyploid and aneuploid (Irvine, 1999; D'Hont and Glaszmann, 2001; Grivet and Arruda, 2002) and have an estimated whole-genome size of 10 Gb (D'Hont and Glaszmann, 2001). An *in situ* hybridization study has shown that the genomes of the commercial hybrids consist of 10–20% chromosomes from *S. spontaneum* and 5–17% recombinant chromosomes between the two species, while the remaining majority of the genome consists of chromosomes from *S. officinarum* (Piperidis and D'Hont, 2001; D'Hont, 2005).

Molecular evidence suggests that polyploid genomes can present dynamic changes in DNA sequences and gene expression, probably in response to genomic shock (genomic remodeling due to the activation of previously deleted heterochromatic elements), and this phenomenon is implicated in epigenetic changes in homologous genes due to intergenomic interactions (McClintock, 1984). The evolutionary success of polyploid species is related to their ability to present greater phenotypic novelty than is observed in their diploid counterparts or even absent in parents (Ramsey and Schemske, 2002). Among other factors, this increase in the capacity for phenotypic variation capacity may be caused by regulation of the allelic dosage (Birchler et al., 2005).

The Brazilian sugarcane variety SP80-3280 is derived from a cross between the varieties SP71-1088 × H57-5028 and is resistant to brown rust caused by *Puccinia melanocephala* (Landell et al., 2005). SP80-3280, which is one of the main Brazilian cultivars (Manechini et al., 2018), was chosen for transcriptome sequencing by SUCEST-FUN (Vettore et al., 2003) and RNAseq (Cardoso-Silva et al., 2014; Nishiyama et al., 2014; Mattiello et al., 2015). Biparental crossing of SP80-3280 has also been used to analyze rust resistance (Balsalobre et al., 2016), quantitative trait loci (QTL) mapping (Costa et al., 2016), and genotyping by sequencing (GBS) (Balsalobre et al., 2017). A Brazilian initiative (Souza et al., 2011) is producing a gene-space genome sequence from SP80-3280, and a draft sugarcane genome based on whole-genome shotgun sequencing was produced (Riaño-Pachón and Mattiello, 2017). Additionally, QTL gene synteny from sorghum has been used to map corresponding bacterial artificial chromosomes (BACs) in SP80-3280 (Mancini et al., 2018).

Three BAC libraries for different sugarcane varieties have been constructed. The oldest one is for the French variety R570 (Tomkins et al., 1999) and contains 103,296 clones with an average insert size of 130 kb, representing 1.2 total genome equivalents. A mix of four individuals derived from the self-fertilization of the elite cultivar R570 (pseudo F2) was reported by Le Cunff et al. (2008) and contains 110,592 clones with an average insert size of 130 kb, representing 1.4x coverage of the whole genome. Additionally, a SP80-3280 library published by Figueira et al. (2012) contains 36,864 clones with an average insert size of 125 kb, representing 0.4 total genome equivalents of coverage.

Sugarcane and sorghum [*Sorghum bicolor* (L.) Moench] share a high level of collinearity, gene structure and sequence conservation. de Setta et al. (2014) contributed to understanding the euchromatic regions from R570 and a few repetitive-rich regions, such as centromeric and ribosomal regions, as well as defining a basic transposable element dataset. The genomic similarity between sugarcane and sorghum has been frequently used to characterize the sugarcane genome (Jannoo et al., 2007; Garsmeur et al., 2011, 2018; Vilela et al., 2017; Mancini et al., 2018), demonstrating the high synteny of sugarcane × sorghum and the high gene structure retention among the different sugarcane homeologs. Additionally, these works contribute to understanding the genomic and evolutionary relationships among important genes in sugarcane using BAC libraries.

Genome organization and expression dynamics are poorly understood in complex polyploid organisms, such as sugarcane, mainly because reconstructing large and complex regions of the genome is a challenge. However, an intriguing question is how such a complex genome can function while handling different copy numbers of genes, different allelic dosages and different ploidies of its homo/homeolog groups. To address this question, we investigated two physically linked genes: an unknown function gene *HP600* with a single copy in the diploid grass group (OrthoDB, Kriventseva et al., 2018) and the gene *CENP-C* (Centromere Protein C, Talbert et al., 2004; Gopalakrishnan et al., 2009; Kato et al., 2013; Sandmann et al., 2017), involved in cell division, localized next to HP600. We examined the genome, transcriptome, evolutionary patterns and genetic interactions/relationships of *HP600* and *CENP-C* in a genomic region from the SP80-3280 sugarcane variety (a *Saccharum* hybrid). First, we defined the genome architecture and evolutionary relationships of *HP600* and *CENP-C* in detail. Second, we used the sugarcane SP80-3280 transcriptome to investigate transcription interactions in each gene (*HP600* and *CENP-C*). Ultimately, we used molecular markers developed from these genes to genotype a segregating population and construct a linkage map and compare it with the physical map.

## Materials and Methods

### Plant Material

The sugarcane varieties were collected from germplasms from the Sugarcane Plant Breeding Program at the active site located in the Agronomic Institute of Campinas (IAC) Sugarcane Center in Ribeirão Preto, São Paulo, Brazil. The youngest leaves from one plant each of SP80-3280 and SPIAC93-3046 were collected from adult plants and immediately stored on dry ice for transportation and finally stored at −80°C until use. These leaves were used for BAC library construction. For the cytogenetic experiments, IACSP95-3018, IACSP93-3046, RB835486, SP80-3280, and SP81-3250 internodes were collected from adult plants. The internodes were placed in cotton soaked in water and left to root. Root tips were collected when they reached 5–15 mm.

### Sequence Analysis and Gene Annotation

The BAC library construction, BAC selection and BAC assembly are described in Supplementary Material. All the BACs were aligned to verify the presence of redundant homeolog sequences. BAC clones with more than 99% similarity were considered the same homeolog. BACs that represented the same homeologs were not combined. The BACs were annotated with the gene prediction programs EUGENE (Sylvain et al., 2008) and Augustus (Keller et al., 2011). The BAC sequences were also searched for genes with BLASTN and BLASTX against the transcripts from the SUCEST-FUN database (http://sucest-fun.org/; Vettore et al., 2003), the CDS of *S. bicolor, Z. mays*, and *O. sativa* from Phytozome v12.0 Goodstein et al., 2012 and the transcripts published by Cardoso-Silva et al. (2014). The BACs were also subjected to BLASTX against *Poaceae* proteins. The candidate genes were manually annotated using *S. bicolor, O. sativa*, and *Z. mays* CDS. The sequences with more than 80% similarity and at least 90% coverage were annotated as genes.

Repetitive content in the BAC clone sequences was identified with the web program LTR_FINDER (Xu and Wang, 2007). Afterward, the BAC sequences were tested by CENSOR (Kohany et al., 2006) against *Poaceae* (Supplementary Material 2).

The phylogenic trees were built by the Neighbor-Joining method (Saitou and Nei, 1987) with nucleic distances calculated with the Jukes-Cantor model (Jukes and Cantor, 1969) in the MEGA 7 software (Kumar et al., 2016). The Kimura 2-parameter (Kimura, 1980) was used as the distance mode.

### Duplication Divergence Time

The gene contents of *HP600* and *CENP-C* in the duplication regions were compared, and the distance “d” for coding regions was determined by Nei-Gojobori with Jukes-Cantor, which is available in the MEGA 7 software (Kumar et al., 2016). The divergence times of the sequences shared by the duplicated regions in the BACs were estimated by T = d/2r. The duplicated sequences were used to calculate the pairwise distances (d), and “r” was replaced by the mutation rate of 6.5 × 10–9 mutations per site per year as proposed by Gaut et al. (1996). For the whole duplication, the distance “d” for noncoding regions was determined with the Kimura 2-parameter model and a mutation rate of 1.3 × 10–8 mutations per site per year as described by Ma and Bennetzen (2004).

The insertion ages of the long terminal repeat (LTR) retrotransposons were estimated based on the accumulated number of substitutions between the two LTRs (d) (SanMiguel et al., 1998) using a mutation rate of 1.3 × 10–8 mutations per site per year as described by Ma and Bennetzen (2004).

### Gene Expression

The transcriptomes of the sugarcane variety SP80-3280 from the roots, shoots and stalks were mapped on *HP600* and *CENP-C* (NCBI SRR7274987). The reads from the sugarcane transcriptomes were mapped to a reference gene with the Bowtie2 software 2.2.5 (Langmead and Salzberg, 2012) with default parameters; low-quality reads and unmapped reads were filtered out (SAMtools -b -F 4), bam files were sorted (SAMtools sort), and only mapped reads to the genes were extracted from the bam files (SAMtools fastq) and recorded in a FASTQ format file.

The resulting reads mapped in the reference gene sequence of *HP600* and *CENP-C* were mapped against each gene haplotype with 100% identity. A haplotype was considered to be expressed when the transcript reads covered the entire gene or mapped exclusively to haplotype SNPs. SNPs not found in the dataset were searched in the SP80-3280 transcriptomes from Vettore et al. (2003), Talbert et al. (2004), and Cardoso-Silva et al. (2014) to verify the SNP presence in transcripts, but they were not used in the expression analysis.

To test whether the haplotypes had the same proportional ratio in the genome and transcriptome, the transcriptomes were mapped against one haplotype of the *HP600* and one of the *CENP-C* with a 90% similarity in Region01. The SNPs found in the transcripts were identified and the coverage and raw variant reads count was used to verify the presence of SNPs not found in BACs. An SNP was considered present in the transcripts if it was represented by at least six transcriptome reads (Kim et al., 2016).

We assumed that one haplotype from each region was missing in the BAC clone data and tested the following two genomic frequencies for comparison with the transcriptome sequences: (1) the missing haplotype had the more common SNP, and (2) the missing haplotype had the variant SNP. When the SNP was not found in the genomic data, we assumed that only the missing haplotype contained the variant SNP.

The frequency of the genomic data was used to test the transcriptome data with R Studio Team (2015) and the exact binomial test [*binom.test*—(Clopper and Pearson, 1934; Conover, 1971; Hollander et al., 1973)]. A *p* ≥ 0.05 is equivalent to a 95% confidence interval for considering the genomic ratio equal to the transcriptome ratio.

### Chromosome Number Determination and BAC-FISH

The chromosome number was performed as described by Guerra (1983) with root tips that were 5–15 mm in length and treated with 5 N HCl for 20 min. The slides were stained with 2% Giemsa for 15 min. Chromosome number was performed for the SP80-3280, SP81-3250, RB83-5486, IACSP95-3018, and IACSP93-3046 varieties. CMA/DAPI coloration was performed by enzymatic digestion as described by Guerra and Souza (2002). The slides were stained with 10 μg/ml DAPI for 30 min and 10 μg/ml CMA for 1 h. Afterwards, the slides were stained with 1:1 glycerol/McIlvaine buffer and visualized.

BAC-FISH was performed using the SP803280 variety. For the mitotic chromosome preparations, root tips that were 5–15 mm in length were collected and treated in the dark with p-dichlorobenzene-saturated solution at room temperature for 2 h, fixed in a freshly prepared 3:1 mixture (ethanol:glacial acetic acid) at 4°C for 24 h and stored at −20°C until use. After being washed in water, the root tips were digested with the following enzyme solution: 2% cellulase (w/v) (Serva, Heidelberg, Baden-Wurtemberg State, Germany), 20% pectinase (v/v) (Sigma, Munich, Baviera State, Germany), and 1% Macerozyme (w/v) (Sigma) at 37°C for 1–2 h (Schwarzacher et al., 1980). The meristems were squashed in a drop of 45% acetic acid and fixed in liquid nitrogen for 15 min. After air-drying, slides with good metaphase chromosome spreads were stored at −20°C.

The Shy064N22 and Shy048L15 BACs, both from the BAC library for the SP80-3280 variety, were used as probes. The probes were labeled with digoxigenin-11-dUTP (Roche) by nick translation. Bacterial artificial chromosome-fluorescence *in situ* hybridization (BAC-FISH) was performed as described by Schwarzacher and Heslop-Harrison (2000) with minor modifications. The *C*_*o*_*t*-100 fraction of the SP80-3280 sugarcane variety genomic DNA, which was used to block repetitive sequences, was prepared according to Zwick et al. (1997). Preparations were counterstained and mounted with 2 μg/ml DAPI in Vectashield (Vector, Burlingame, CA, USA).

The sugarcane metaphase chromosomes were observed and photographed, depending on the procedure, with transmitted light or epifluorescence under an Olympus BX61 microscope equipped with the appropriate filter sets (Olympus, Shinjuku-ku, Tokyo, Japan) and a JAI® CV-M4 + CL monochromatic digital camera (JAI, Barrington, N.J., USA). Digital images were imported into Photoshop 7.0 (Adobe, San Jose, Calif., USA) for pseudocoloration and final processing.

### Genetic Map Construction

The BAC haplotypes were used to identify 44 sugarcane SNPs in the *HP600* and *CENP-C* exons. The SNP genotyping method was based on MALDI-TOF analysis performed on a mass spectrometer platform from Sequenom Inc.,® as described by Garcia et al. (2013). The mapping population consisted of 151 full siblings derived from a cross between the SP80-3280 (female parent) and RB835486 (male parent) sugarcane cultivars, and the genetic map was constructed as described by Balsalobre et al. (2017) using SuperMASSA software (Serang et al., 2012). The SuperMASSA software calculates all possible ploidies for a locus and produces the most likely ploidy.

## Results

### Relationship Between Region01 and Region02

**Figure 1:**
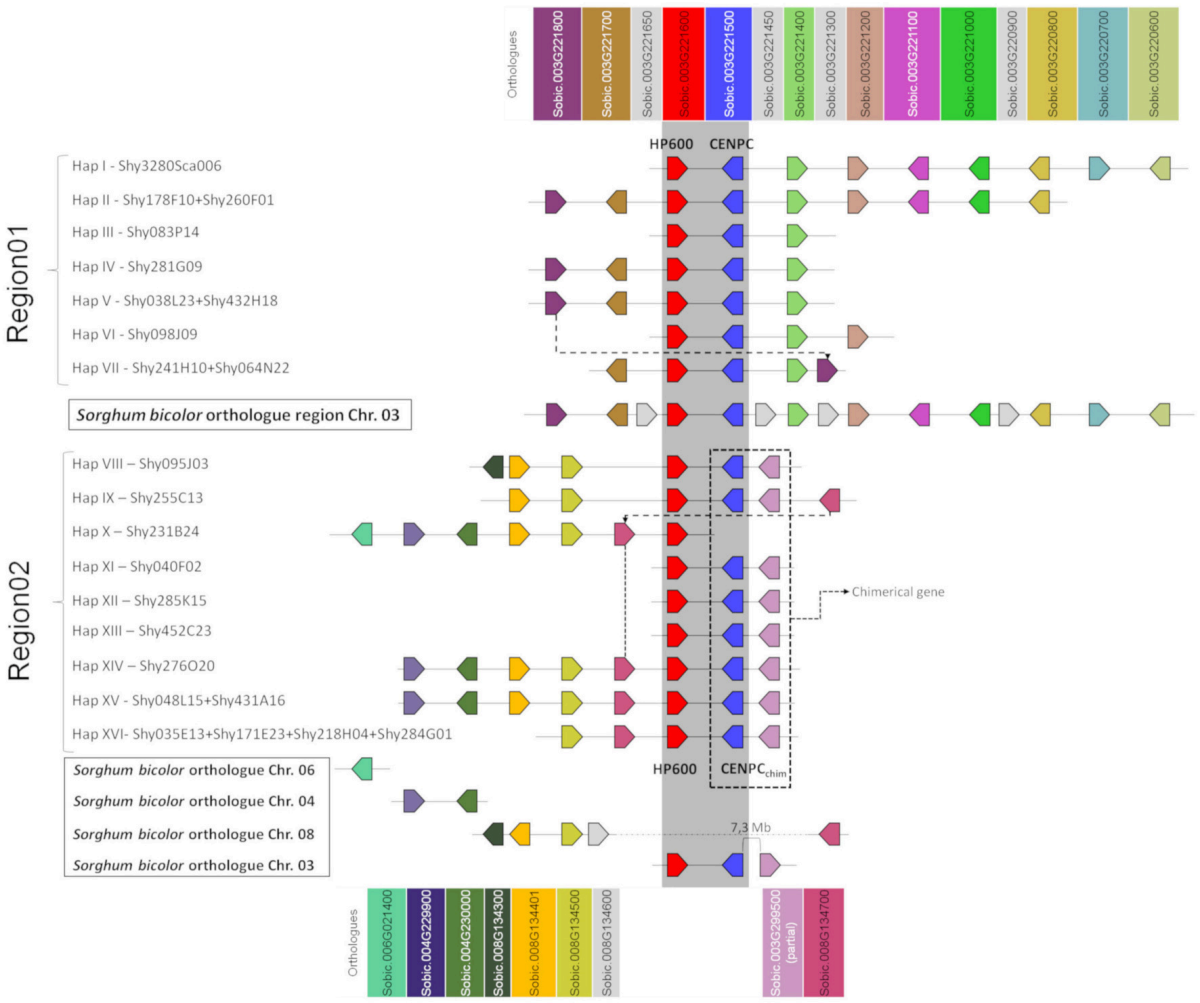
Schematic representation of the sugarcane BAC haplotypes from Region01 and Region02. Squares of the same color represent sugarcane genes orthologous to *Sorghum bicolor* genes. Dotted lines connect the homologous genes in sugarcane at different positions. In sugarcane Region02, the *CENP-C* haplotypes in Region02 are represented by two squares (blue and pink), where each square represents a partial gene fusion. The dark gray strip represents the shared region from Region01 and Region02 (duplication). The genes in light gray (from *S. bicolor*) are not found in the sugarcane BACs. The representation is not to scale. The orientation of transcription is indicated by the direction of the arrow at the end of each gene.

Annotation of *HP600* and *CENP-C* in the 16 BAC haplotypes revealed two groups of BACs. One group had the expected exon/intron organization compared with *S. bicolor HP600* (five exons in sorghum) and *CENP-C* (14 exons in sorghum). This region was further designated as Region01 (see Supplementary Table 1, Supplementary Material - 10 BACs and 7 haplotypes—Figure 1 - haplotype I to haplotype VII). The other group was found to have fewer exons than expected (compared with *S. bicolor*) for both *HP600* and *CENP-C* and was designated Region02 (see Supplementary Table 1, Supplementary Material−13 BACs and 9 haplotypes—Figure 1 - haplotype VIII to haplotype XVI).

A comparison of the BAC haplotypes from Region01 and Region02 revealed an 8-kb shared region in sugarcane. The 8-kb duplication spanned from the last three exons of *HP600* to the last seven exons of *CENP-C*. *HP600* and *CENP-C* were physically linked, but the orientation of the genes was opposite (see Supplementary Figure 2B). A phylogenetic tree was constructed to examine the relationships among this 8-kb region (see Supplementary Figure 2A). The orthologous region from *S. bicolor* was used as an outgroup, and the separation in the two groups (Region01 and Region02) suggests that the shared 8-kb sequence appeared as a consequence of a sugarcane-specific duplication.

**Figure 2:**
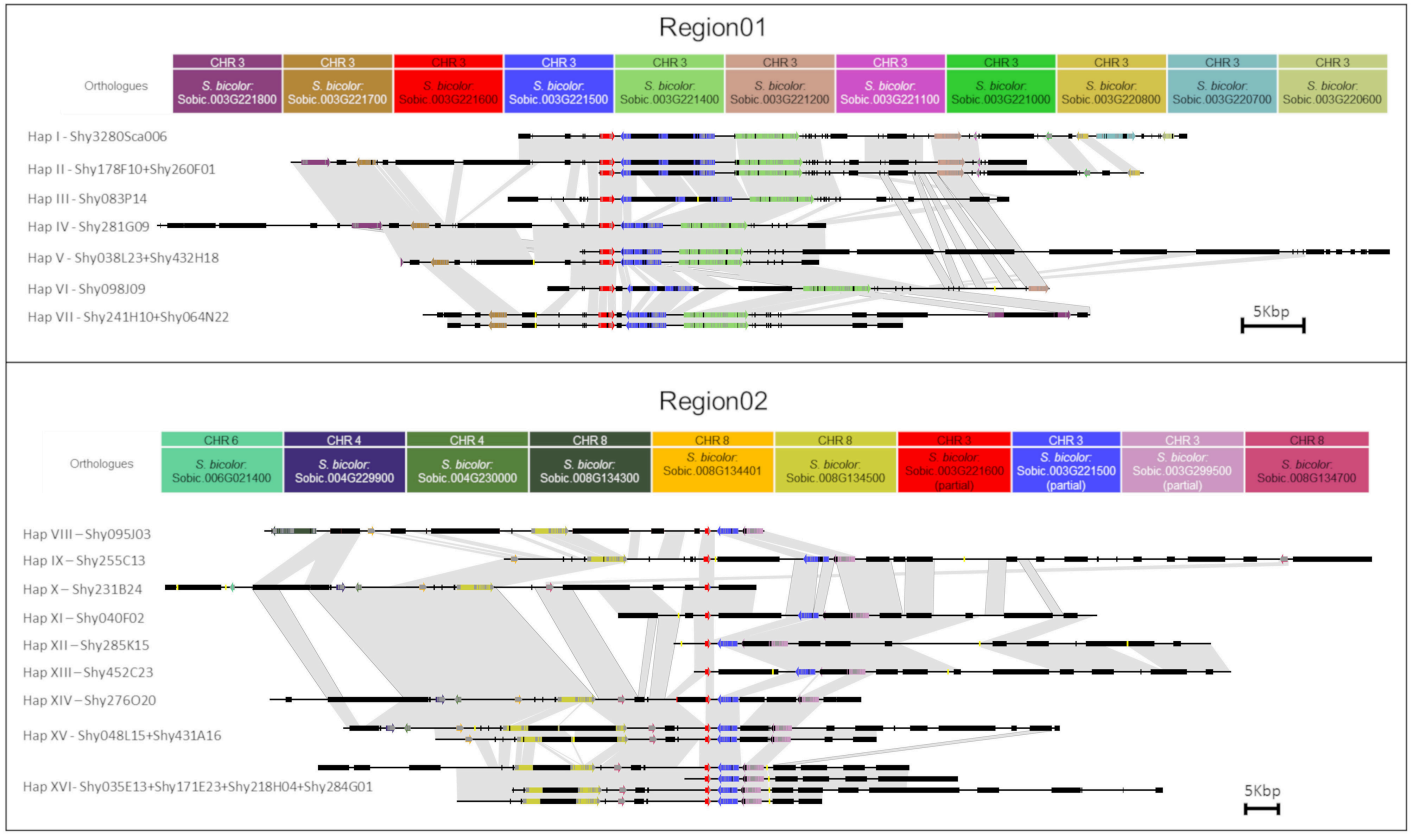
Representation of each sugarcane BAC from Region01 and Region02. Arrows and rectangles of the same color represent the homologous genes in sugarcane. Black rectangles represent repeat regions. Yellow lines represent gaps. Similar regions are represented by a gray shadow connecting the BACs. The orientation of transcription is indicated by the direction of the arrow at the end of each gene. Scale representation.

Region01 BACs exhibited high gene collinearity with *S. bicolor*. However, in the BAC haplotype VII, a change in gene order involving the sorghum orthologs Sobic.003G221800 and Sobic.003G221400 was observed (Figure 1, dotted line). Sobic.003G221800 is missing in this position from haplotypes I, II, and VI. Region01 and Region02, except for the genes *HP600* and *CENP-C*, contain different sorghum orthologous genes (Figure 1). Region02 was found to be non-collinear with *S. bicolor* (Figures 1, 2), which reinforces the notion of a specific duplication in sugarcane. Region02 appeared as a mosaic formed by different sorghum orthologous genes distributed in different chromosomes and arose by duplication after the separation of sorghum and sugarcane.

In Region02, the Sobic.008G134300 orthologous gene was found only in haplotype VIII, and the Sobic.008G134700 ortholog was found in a different position in haplotype IX (Figure 1, dotted line in Region02 and Figure 2). The phylogenetic analysis of Sobic.008G134700 and sugarcane orthologs demonstrated that sugarcane haplotype IX is more closely related to sorghum than to other sugarcane homeologs (see Supplementary Figure 3). Additionally, the orientation of transcription of the Sobic.008G134700 ortholog in haplotype IX is opposite that of the other sugarcane haplotypes (Figures 1, 2). This finding suggests that this gene could be duplicated (paralogs) or translocated (orthologs) in haplotypes X, XIV, XV, and XVI. No *S. bicolor* orthologous region that originated from Region02 could be determined, as it contained genes from multiple sorghum chromosomes.

Twenty LTR retrotransposons were located in the two regions, but no LTR retrotransposons were shared among the haplotypes from Region01 and Region02. The oldest LTR retrotransposon insertions were dated from 2.3 Mya (from haplotype VIII from Region02, a DNA/MuDR transposon, similar to MUDR1N_SB). Four LTR retrotransposons, localized in the non-shared duplicated region similar to RLG_scAle_1_1-LTR, had identical sequences (Region01: Sh083P14_TE0360—haplotype III and Sh040F02_TE0180—haplotype XI; Region02: Sh285K15_TE0060—haplotype XII and Sh452C23_TE0090—haplotype XIII).

To estimate the genomic diversity in sugarcane haplotypes from both regions (analyzed together and separately), the shared 8-kb region (duplication) was used (see Supplementary Table 4), and the SNPs were identified. The diversity in the *HP600* and *CENP-C* genes was analyzed, and one SNP was observed every 43 bases (Region02) and 70 bases (Region01). We searched for SNPs that could distinguish each region (see Supplementary Table 5) in the *HP600* and *CENP-C* genes, and one SNP was found for every 56 bases (20 SNPs in total). Additionally, small (3–10 bases) and large (30–200 bases) insertions were found.

### *HP600* and *CENP-C* Haplotypes and Phylogenetics

Gene haplotypes, i.e., genes with the same coding sequences (CDSs), from *HP600* and *CENP-C* that have the same coding sequence (i.e., exons) in different BAC haplotypes were considered the same gene haplotype. In Region01, four haplotypes of *HP600* were identified. In sorghum, the size of *HP600* is 187 amino acids (561 base pairs). *HP600* has two different sizes in sugarcane haplotypes: one of 188 amino acids (564 base pairs—haplotype I/II/VI, haplotype IV/V and haplotype VII) and another of 120 amino acids (360 base pairs—haplotype III). The *HP600* haplotype III has a base deletion at position 77, causing a frameshift that results in a premature stop codon.

In Region02, *HP600* exhibited the following six haplotypes: haplotype VIII, haplotype IX, haplotype X/XI/XII/XIII/XIV, haplotype XV, and haplotype XVI. *HP600* haplotype IX carried an insertion of eight bases in the last exon that caused a frameshift.

In *S. bicolor, CENP-C* is formed by 14 exons (Talbert et al., 2004) encoding 694 amino acids (2,082 base pairs). In sugarcane, the haplotypes from Region01 had 14 exons that gave rise to a 708 or 709 amino acid (2,124 or 2,127 bases) protein. Talbert et al. (2004) described two haplotypes in sugarcane EST clones (Vettore et al., 2003), *CENP-C1*, and *CENP-C*2, which correspond to haplotypes I/II and IV/V, respectively. In addition to CENP-C1 and CENP-C2, three other *CENP-C* haplotypes were observed, including haplotype III, haplotype VI, and haplotype VIII.

In Region02, the sugarcane duplication of *CENP-C* consisted of the last seven exons (exons 8–14 from *CENP-C* in Region01), and the following six haplotypes were found: haplotype VIII, haplotype IX, haplotypes XI/XII/XIII, haplotype XIV, haplotype XV, and haplotype XVI. The haplotype X BAC sequence finished before the CENP-C gene (Figure 1).

To reconstruct a phylogenetic tree for *HP600* and *CENP-C* from both regions, the orthologs from *O. sativa* and *Zea mays* L. were searched. The rice *HP600* and *CENP-C* orthologs, LOC_Os01g43060 and LOC_Os01g43050, respectively, were recovered. Maize has gone through tetraploidization since its divergence from sorghum ~12 million years ago (Woodhouse et al., 2010). The maize *HP600* ortholog search returned the following three possible genes with high similarity: GRMZM2G114380 (chromosome 03), GRMZM2G018417 (chromosome 01), and GRMZM2G056377 (chromosome 01). The *CENP-C* maize ortholog search returned the following three possible genes with high similarity: GRMZM2G114315 (chromosome 03), GRMZM2G134183 (chromosome 03), and GRMZM2G369014 (chromosome 01).

Two phylogenetic trees were constructed (see Supplementary Figure 4), one for *HP600* (see Supplementary Figure 4A) and the other for *CENP-C* (see Supplementary Figure 4B), using sugarcane *HP600* and *CENP-C* haplotypes from both regions. The results demonstrated that the haplotypes from Region01 and Region02 are more similar to themselves than they are to those of sorghum or rice.

The divergence times among sugarcane *HP600* haplotypes and sorghum ranged from 1.5 to 4.5 Mya. For *CENP-C*, the haplotype divergence time rates were 0.3–0.7 Mya, and the comparison with sorghum indicated 4.2–4.5 Mya for the highest values. The estimated sugarcane x sorghum divergence time was 5 Mya (Ming et al., 1998) to 8–9 Mya (Jannoo et al., 2007; Zhang et al., 2018a).

### Chromosome Number Determination and BAC-FISH

The determination of the range of *CENP-C* and *HP600* loci that are present in the sugarcane genome was performed using *in situ* hybridization. First, the number of chromosomes in the SP80-3280 sugarcane variety was defined, but the number of clear and well-spread metaphases for the SP80-3280 variety was < 10 for each chromosome number (see Supplementary Table 6). We expanded the analysis to four more sugarcane varieties (SP81-3250, RB835486, IACSP95-3018, and IACSP93-3046) to improve the conclusions (see Supplementary Figures 5A–E and Supplementary Table 6). The most abundant number of chromosomes was 2n = 112 (range: 2n = 98–2n = 118 chromosomes). The chromosome number for the *Saccharum* hybrid cultivar SP80-3280 was found to be 2n = 112 (range: 2n = 108–2n = 118 chromosomes—see Supplementary Table 6). Vieira et al. (2018) also identified 2n = 112 chromosomes in the IACSP93-3046 variety.

As a second step, we used two varieties with the best chromosome spreads, i.e., IACSP93-3046 and IACSP95-3018, for the CMA/DAPI banding patterns (see Supplementary Figures 5F–I). The IACSP93-3046 variety exhibited at least six terminal CMA^+^/DAPI^−^ bands, one chromosome with CMA^+^/DAPI° and two chromosomes with adjacent intercalations of CMA^+^ and DAPI^+^ in the same chromosome (see Supplementary Figures 5F,G). The IACSP95-3018 variety revealed seven terminal CMA^+^/DAPI^−^ bands, and at least two chromosomes exhibited adjacent CMA^+^ and DAPI^+^, one of which was in the intercalary position and the other was in the terminal position (see Supplementary Figures 5H,I).

**Figure 3:**
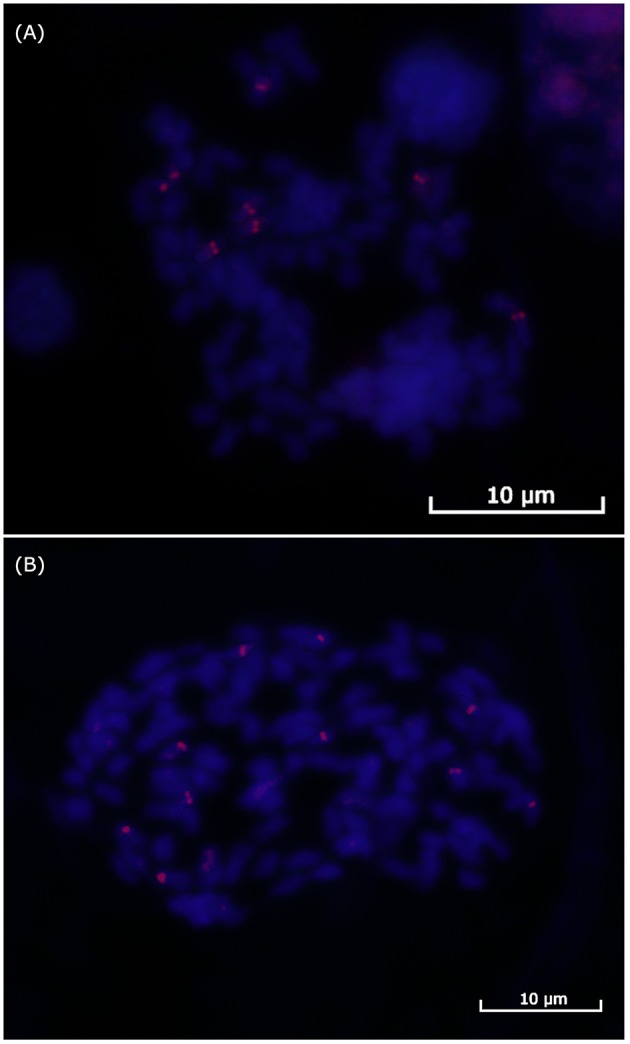
FISH of the sugarcane BACs. **(A)** BAC Shy065N22 hybridization in sugarcane variety SP-803280 mitosis showing eight signals for Region01. **(B)** BAC Shy048L15 hybridization in sugarcane variety SP-803280 mitosis showing 10 signals for Region02.

Finally, we performed BAC-FISH in the better metaphases from the SP80-3280 variety using Shy064N22 (haplotype VII) from Region01; 64 metaphases with some signal of hybridization were obtained, while 69 were obtained for the BAC-FISH of Shy048L15 (haplotype XI) from Region02. At least six metaphases for each region were used to determine the number of signals. For BAC Shy064N22 Region01, eight signals could be counted (Figure 3A), and for BAC Shy048L15 in Region02, 10 signals could be defined (Figure 3B).

The results observed so far suggest differences between the haplotypes, i.e., different TEs, insertions and even gene insertions/translocations. We used an identity of 99% to determine the presence of the same BAC haplotype. The possibility of haplotypes with more than 99% similarity *in vivo* could not be tested with our data, since it is not possible distinguish a mismatch in a sequence assembly from a real haplotype.

### Expression of *HP600* and *CENP-C* Haplotypes

The transcriptomes of the SP80-3280 sugarcane variety from the roots, shoots, and stalks were mapped on *HP600* and *CENP-C* (NCBI SRR7274987), and the set of transcripts was used for the transcription analyses. All of the *HP600* haplotypes from Region01 were covered by the reads, including haplotype III with a premature stop codon. None of the *HP600* haplotypes from Region02 were found, suggesting that *HP600* is not expressed from Region02 (see Supplementary Figure 2).

For the *CENP-C* gene from Region01, haplotypes IV/V were found to be expressed. Furthermore, haplotypes I/II, haplotype VI and haplotype VII were fully covered by the reads, except for the first three SNPs, but these SNPs were described in the work of Talbert et al. (2004) under the *CENP-C*1 haplotype, suggesting that the set of reads did not cover this region. For haplotype III, one SNP was not found, but nine exclusive SNPs from this haplotype were represented. Therefore, all *CENP-C* haplotypes from Region01 were considered to be expressed.

The *CENP-C* haplotypes I/II, III, and VI from Region01 have large retrotransposons in the introns (Figure 2—black rectangles). Additionally, no evidence of substantial influence on expression could be found for this gene, which may indicate the silencing of these LTR retrotransposons, as discussed by Kim and Zilberman (2014).

**Figure 4:**
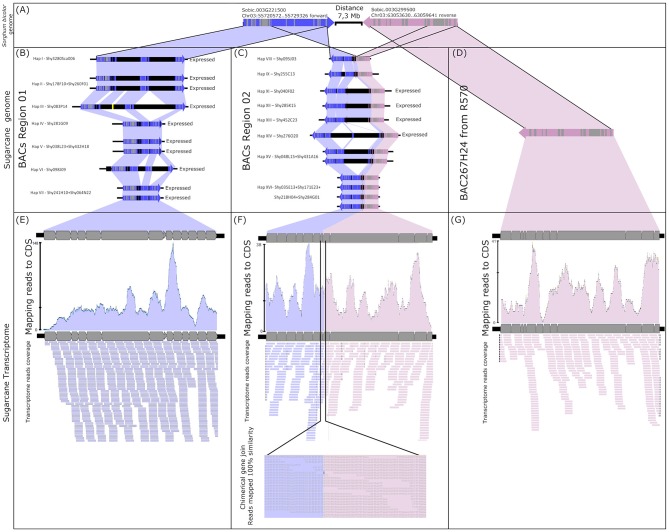
Fusion gene formation of *CENP-C* and Sobic003G299500. **(A)** Sorghum *CENP-C* and Sobic003G299500 genome location. **(B)** Sugarcane genomic *CENP-C* haplotypes in Region01 (all expressed). **(C)** Partially duplicated sugarcane paralogs of *CENP-C* and Sobic003G299500 haplotypes in Region02 (only haplotypes XI/XII/XIII and haplotype XIV have evidence of expression). **(D)** Sugarcane ortholog of Sobic003G299500 found in the sugarcane R570 BAC library. **(E)** Transcripts from sugarcane SP80-3280 mapped against the CDS of sugarcane *CENP-C* haplotypes from Region01. **(F)** Transcripts from sugarcane SP80-3280 mapped against the sugarcane chimeric paralogs of *CENP-C* and Sobic003G299500. As evidence of fusion gene formation, the transcripts show the fusion point of the paralogs. **(G)** Transcripts from sugarcane SP80-3280 mapped against the CDS of the sugarcane R570 Sobic003G299500 ortholog.

The mapping of the transcript reads in the *CENP-C* haplotypes from Region02 revealed evidence of a chimeric gene (Figure 1, dotted rectangle, and Figure 4). The chimeric gene was formed by the first five exons from the Sobic.003G299500 sugarcane orthologous gene and the eighth to fourteenth exons from *CENP-C* (Figure 4C). RNAseq reads overlapped the region corresponding to the union of the chimeric gene (position 1253 from the *CENP-C* haplotypes from Region02 by 38 reads; Figure 4F).

The sugarcane gene orthologous to Sobic.003G299500 was represented by BAC BAC267H24 (GenBank KF184671) from the sugarcane hybrid R570 as published by de Setta et al. (2014) under the name “SHCRBa_267_H24_F_10” (Figure 4D). This finding indicated that the ancestral genes from sorghum (orthologs) were retained in the sugarcane genome (Figures 4B–D) and that the chimeric gene was formed by the fusion of a partial duplication of *CENP-C* and the sorghum ortholog gene Sobic.003G299500 (Figure 4C).

Two chimeric *CENP-C* haplotypes from Region02 were fully mapped with transcripts, i.e., haplotypes XI/XII/XIII and haplotype XIV. The chimeric *CENP-C* haplotypes IX and XVI from Region02 were not fully mapped, but exclusive SNPs from these haplotypes were recovered. The *CENP-C* haplotypes VIII and XV from Region02 exhibited no exclusive SNPs in the transcriptome, and evidence for the expression of these two haplotypes remains undefined.

### Comparison With Other *Saccharum* Genomes

A search for the *HP600* and *CENP-C* genes against the sugarcane R570 mosaic monoploid genome (Garsmeur et al., 2018) returned no hits, indicating that both genes were not represented in the R570 BACs. Comparisons of the BAC sequences against the sugarcane SP80-3280 genome draft using BLASTN (Riaño-Pachón and Mattiello, 2017) resulted in matches within gene regions, but no genome contig covered a whole BAC, and the BAC TEs matched with several genome contigs (see Supplementary Figure 6). The matches with gene regions provide further support for our assembly process.

A BLAST search of the all genes recovered from Region01 and Region02 against the *S. spontaneum* genome (Zhang et al., 2018b) resulted in the recovery of the chromosome of each gene in *S. spontaneum* (see Supplementary Tables 2, 3). *HP600* was found in chromosomes Chr2D, Chr3B, Chr3C and Chr3D from *S. spontaneum*. In chromosome Chr2D, *HP600* was found as in Region02 BACs, and in chromosomes Chr3B and Chr3C, as in Region02 BACs. In chromosome Chr3D, *HP600* was duplicated at positions 14833330 and 35428849, both of which had the same architecture as in Region01 (five exons).

Both the *CENP-C* and chimeric *CENP-C* sequences were used to search for the *CENP-C* gene. The *CENP-C* gene was found in *S. spontaneum* chromosomes Chr3B and Chr3C. Chromosome Chr3D had a duplication of *CENP-C* at position 14835786 (complete gene) and position 35431299 (partial, last six exons—not found in our data). In chromosome Chr7B, the 9 first exons were found, but this architecture was not found in our data. The chimeric *CENP-C* gene was found in chromosomes Chr2A and Chr2D.

Regarding these results, Region01 is present in Chr03B, Chr3C, and Chr3D (only in position 14835786), with *HP600* and *CENP-C* physically side by side (see Supplementary Figure 7). Region02 is only represented in chromosome Chr02D with a duplication composed of *HP600* and the *CENP-C* chimera physically side by side. Another copy of the *CENP-C* chimera was found in chromosome Chr2A, but without the presence of *HP600*. Additionally, the Sobic.003G299500 ortholog gene, which was fused with *CENP-C*, was also found with its complete sequence (as demonstrated in Figure 4D) in chromosome Chr3A at position 16992405 and duplicated in two positions, 32628152 and 60347125, in chromosome Chr3C.

### How the Locus Number of Homeologs Influences Expression

We searched the SNPs in the BAC sequences and RNAseq reads (i.e., only in the transcriptome of the SP80-3280 sugarcane variety from the roots, shoots and stalks—NCBI SRR7274987) and compared the correspondences to the *HP600* and *CENP-C* genes. For Region01 and Region02, we defined the ploidies as 8 and 10, respectively, based on the BAC-FISH data. The numbers of BAC haplotypes recovered for Region01 and Region02 were seven and nine, respectively, which indicated one missing BAC haplotype in each region.

The missing BAC haplotypes were determined by searching for SNPs present only in the transcriptome. For the *HP600* haplotypes from Region01 (Table 1), six SNPs were found in the transcriptome and not in the BAC haplotypes, including a (GAG)3 -> (GAG)2 deletion. For the *CENP-C* gene (Table 2), eight SNPs were not represented in the genomic haplotypes. The presence of SNPs only in the transcript data corroborates the assumption that (at least) one genomic haplotype was missing in each region.

**Table 1:** Genomic frequencies of the SNPs in the *HP600* haplotypes in Region01.

**SNP**	**Name**	**Change**	**Polymorphism type**	**Position**	**Coverage**	**Variant coverage**	**Genomic detected**	**Transcriptome proportion**	**Missing haplotype for more common SNP**				**Missing haplotype for variant SNP**			
									**Genomic variant**	**Genomic**	**Genomic proportion**	***P*-value (binomial test)**	**Genomic variant**	**Genomic**	**Genomic proportion**	***P*-value (binomial test)**
1	C	G -> C	SNP (transversion)	12	443	101	Yes	0.23	1	7	0.125	2.32E-09	2	6	0.25	2.98E-01^*^
2	–	-C	Deletion	78	515	28	Yes	0.05	1	7	0.125	1.13E-07	2	6	0.25	4.76E-32
3	T	C -> T	SNP (transition)	133	542	38	Yes	0.07	1	7	0.125	5.16E-05	2	6	0.25	1.62E-27
4	A	G -> A	SNP (transition)	153	577	33	Yes	0.06	1	7	0.125	9.76E-08	2	6	0.25	1.56E-34
5	TT	GG -> TT	Substitution	166	699	137	Yes	0.2	1	7	0.125	1.18E-07	2	6	0.25	8.85E-04
6	T	C -> T	SNP (transition)	263	569	55	No	0.1	1	7	0.125	4.23E-02	1	7	0.125	4.23E-02
7		(GAG)3 -> (GAG)2	Deletion (tandem repeat)	283	654	42	No	0.06	1	7	0.125	4.35E-07	1	7	0.125	4.35E-07
8	C	T -> C	SNP (transition)	429	849	83	No	0.1	1	7	0.125	1.68E-02	1	7	0.125	1.68E-02
9	A	G -> A	SNP (transition)	434	993	69	No	0.07	1	7	0.125	1.68E-08	1	7	0.125	1.68E-08
10	C	G -> C	SNP (transversion)	436	1035	275	Yes	0.27	2	6	0.25	2.51E-01^*^	3	5	0.375	1.196E-13
11	T	G -> T	SNP (transversion)	463	936	56	No	0.06	1	7	0.125	5.11E-11	1	7	0.125	5.11E-11
12	A	C -> A	SNP (transversion)	519	679	57	No	0.08	1	7	0.125	9.10E-04	1	7	0.125	9.10E-04

Genome and transcriptome SNPs were used. The global expression (in diverse tissues) was used to determine whether the genomic frequency could explain the transcription frequency (H_0_). The binomial test was used to verify H_0_. The

“*” *in p-values reflect the acceptance of H_0_*.

**Table 2:** Genomic frequencies of the SNPs in the *CENP-C* haplotypes in Region01 and Region02.

**SNP**	**Name**	**Change**	**Polymorphism type**	**Position**	**Coverage**	**Variant coverage**	**Genomic detected**	**Transcriptome proportion**	**Missing haplotype for more common SNP**				**Missing haplotype for variant SNP**			
									**Genomic variant**	**Genomic**	**Genomic proportion**	***P*-value (binomial test)**	**Genomic variant**	**Genomic**	**Genomic proportion**	***P*-value (binomial test)**
1	G	C -> G	SNP (transversion)	106	16	13	Yes	0.81	5	3	0.63	1.95E-01^*^	4	4	0.5	2.13E-02
2	G	A -> G	SNP (transition)	150	19	8	Yes	0.42	1	7	0.13	1.25E-03	2	6	0.25	1.08E-01^*^
3	C	G -> C	SNP (transversion)	246	34	7	Yes	0.21	1	7	0.13	1.87E-01^*^	2	6	0.25	6.93E-01^*^
4	T	A -> T	SNP (transversion)	369	65	7	Yes	0.11	1	7	0.13	8.51E-01^*^	2	6	0.25	6.14E-03
5	A	G -> A	SNP (transition)	371	68	19	No	0.28	1	7	0.13	6.21E-04	1	7	0.13	6.21E-04
6	C	T -> C	SNP (transition)	390	64	15	No	0.23	1	7	0.13	1.32E-02	1	7	0.13	1.32E-02
7	G	T -> G	SNP (transversion)	513	46	12	Yes	0.26	3	5	0.38	1.28E-01^*^	4	4	0.5	1.64E-03
8	A	G -> A	SNP (transition)	518	45	10	Yes	0.22	2	6	0.25	7.34E-01^*^	3	5	0,375	4.40E-02
9	T	G -> T	SNP (transversion)	731	54	8	Yes	0.15	2	6	0.25	1.14E-01^*^	3	5	0,375	3.58E-04
10	C	A -> C	SNP (transversion)	1008	56	9	No	0.16	1	7	0.13	4.17E-01^*^	1	7	0.13	4.17E-01^*^
11	T	C -> T	SNP (transition)	1061	91	29	Yes	0.32	2	6	0.25	1.46E-01^*^	3	5	0,375	2.81E-01^*^
12	T	C -> T	SNP (transition)	1088	77	41	Yes	0.53	4	4	0.50	6.48E-01^*^	3	5	0,375	6.37E-03
13	T	C -> T	SNP (transition)	1190	76	9	Yes	0.12	2	6	0.25	7.49E-03	3	5	0,375	1.10E-06
14	A	G -> A	SNP (transition)	1209	76	20	No	0.26	1	7	0.13	1.31E-03	1	7	0.13	1.31E-03
15	T	A -> T	SNP (transversion)	1251	62	10	Yes	0.16	2	6	0.25	1.41E-01^*^	3	5	0,375	3.29E-04
16	G	A -> G	SNP (transition)	1255	62	55	Yes	0.89	6	2	0.75	1.19E-02	5	3	0,625	5.15E-06
17		-ATG	Deletion	1307	75	9	Yes	0.12	1	7	0.13	1.00E+00^*^	2	6	0.25	7.38E-03
18	G	A -> G	SNP (transition)	1314	90	23	Yes	0.26	1	7	0.13	6.50E-04	2	6	0.25	9.03E-01^*^
19	G	T -> G	SNP (transversion)	1347	103	13	Yes	0.13	2	6	0.25	2.88E-03	3	5	0,375	3.09E-08
20	A	T -> A	SNP (transversion)	1384	101	37	Yes	0.37	1	7	0.13	5.30E-10	2	6	0.25	1.09E-02
21	G	C -> G	SNP (transversion)	1424	80	9	No	0.11	1	7	0.13	8.66E-01^*^	1	7	0.13	8.66E-01^*^
22	A	C -> A	SNP (transversion)	1437	84	10	Yes	0.12	1	7	0.13	1.00E+00^*^	2	6	0.25	5.12E-03
23	TT	AA -> TT	Substitution	1481	62	7	No	0.11	1	7	0.13	1.00E+00^*^	1	7	0.13	1.00E+00^*^
24	G	A -> G	SNP (transition)	1527	106	90	Yes (duplication)	0.85								
25	C	T -> C	SNP (transition)	1540	139	86	Yes (duplication)	0.62								
26	A	T -> A	SNP (transversion)	1584	253	235	Yes (duplication)	0.93								
27	A	G -> A	SNP (transition)	1638	247	39	Yes (duplication)	0.16								
28	C	A -> C	SNP (transversion)	1648	209	106	Yes (duplication)	0.51								
29	A	C -> A	SNP (transversion)	1739	122	16	Yes (duplication)	0.13								
30	T	C -> T	SNP (transition)	1751	132	32	Yes (duplication)	0.24								
31	A	G -> A	SNP (transition)	1753	138	16	Yes (duplication)	0.12								
32	A	C -> A	SNP (transversion)	1762	131	21	No (duplication)	0.16								
33	T	A -> T	SNP (transversion)	1776	125	75	Yes (duplication)	0.6								
34	C	G -> C	SNP (transversion)	1796	88	31	No (duplication)	0.35								
35	G	C -> G	SNP (transversion)	1808	37	25	Yes	0.68	4	3	0.57	0.00E+00^*^	4	4	0.57	8.90E-01^*^
36	T	C -> T	SNP (transition)	1808	78	41	Yes (duplication)	0.53								
37	T	C -> T	SNP (transition)	1814	78	27	Yes (duplication)	0.35								
38	T	C -> T	SNP (transition)	1827	68	7	Yes (duplication)	0.1								
39	A	T -> A	SNP (transversion)	1830	65	8	Yes (duplication)	0.12								
40	A	G -> A	SNP (transition)	1839	62	23	Yes (duplication)	0.37								
41	A	G -> A	SNP (transition)	1853	52	6	Yes (duplication)	0.12								
42	C	A -> C	SNP (transversion)	1866	47	30	Yes (duplication)	0.64								
43	A	C -> A	SNP (transversion)	1910	152	34	Yes (duplication)	0.22								
44	A	G -> A	SNP (transition)	1917	158	103	Yes (duplication)	0.65								
45	G	T -> G	SNP (transversion)	1922	165	110	Yes (duplication)	0.67								
46	T	A -> T	SNP (transversion)	1938	170	41	Yes (duplication)	0.24								
47	A	C -> A	SNP (transversion)	2039	196	37	Yes (duplication)	0.19								
48	T	C -> T	SNP (transition)	2043	196	143	Yes (duplication)	0.73								
49	G	T -> G	SNP (transversion)	2080	177	88	Yes (duplication)	0.5								
50	C	A -> C	SNP (transversion)	2123	126	89	Yes (duplication)	0.71								

Genome and transcriptome SNPs were used. The global expression (in diverse tissues) was used to determine whether the genomic frequency could explain the transcription frequency (H_0_). The binomial test was used to verify H_0_. The

“*” *in p-values reflect the acceptance of H_0_*.

Using the results obtained from the RNAseq mapping of the haplotypes, we also assumed that all haplotypes for the *HP600* gene were expressed in Region01 and that none were expressed in Region02. For *CENP-C*, all haplotypes from Region01 were considered expressed, and it was not possible to identify how many haplotypes were expressed in Region02 (chimeric gene); thus, we used only the non-duplicated portion of *CENP-C* (exons one to seven from the *CENP-C* gene).

We formed the following three assumptions using the previous results: (I) there is a missing haplotype for each region; (II) all *HP600* haplotypes from Region01 are expressed, and there is no expression of *HP600* in Region02; and (III) *CENP-C* is expressed in both regions, but it is only possible to infer that all haplotypes are expressed in Region01. Using these premises, we investigated the possibilities of the genome SNP ratio (or BAC haplotype) being expressed as the transcript SNP ratio. Therefore, if the haplotypes contribute equally to expression, one SNP found in a BAC should have the same ratio (dosage) for the transcriptome data. Since we found evidence for a missing haplotype, the following two tests were performed: (I) we determined whether the missing BAC haplotype contributed to the dosage of more common SNPs and (II) we determined whether the missing BAC haplotype contributed to the dosage of the variant SNP.

For the *HP600* haplotypes from Region01 (Table 1), only SNPs 10 and 1 had significant *p*-values for hypotheses (I) and (II), respectively. These results suggested that the BAC haplotype ratio does not explain the transcriptome ratio. The transcript frequencies of SNPs 2, 3, and 4 (Table 1) were < 0.125 (the minimum expected ratio for 1:7). To explain these frequencies, the dosage of the SNPs should be higher than a ploidy of eight (i.e., more than twelve), and our data do not support this possibility. The three variant SNPs came from *HP600* haplotype III. This finding could be evidence of some differential expression of the gene haplotypes, which could suggest that haplotype III is expressed at a lower level than the others for the *HP600* gene.

For *CENP-C*, only the non-duplicated portions of the haplotypes from Region01 were used. At least one hypothesis was accepted for 17 (70%) SNPs (Table 2). The mean coverage of the SNPs was 64 reads per SNP, which could be considered low coverage when an eight-ploidy region (Region01) is being inspected (Table 2). Moreover, the result suggests that the haplotypes from Region01 are equally expressed.

### Genetic Mapping

**Figure 5:**
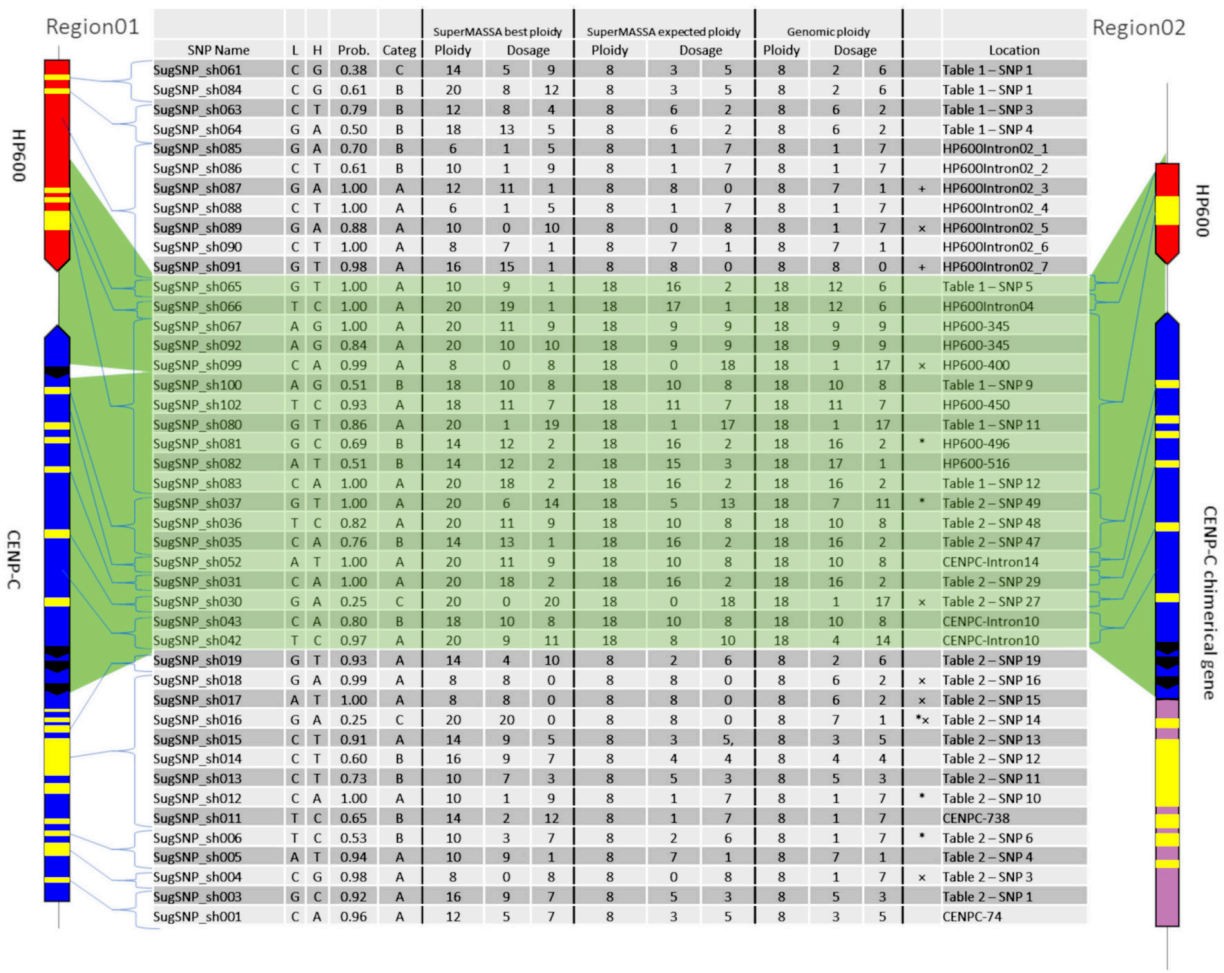
Ploidy and dosage in the sugarcane genomic DNA (BACs) and the SuperMASSA estimation. The location of each SNP is shown by one haplotype from Region01 and one haplotype from Region02. “SuperMASSA Best Ploidy” means the SuperMASSA best ploidy with a posteriori probability >0.8. “SuperMASSA Expected Ploidy” means we fixed the ploidy of the loci in SuperMASSA according to the BAC-FISH and BAC sequencing results. “Genomic Ploidy” means the ploidy of the loci according to the BAC-FISH and BAC sequencing results. “^*^” means the SNP was found only in the transcriptome.

For the genetic mapping, 44 SNPs (see Supplementary Table 7) were used to develop molecular markers (Figure 5) and construct a genetic map. The markers from introns and exons were drawn along Region01 (Figure 5, “Location” column), including the duplicated region found in Region02. Among them, seven exhibited no variants presence in genotyping (Figure 5—“ × ” marked), but five were detected in the RNAseq reads. Two markers (Figure 5—“+” marked) were only detected for the “SuperMASSA best ploidy,” which was a ploidy higher than the “SuperMASSA expected ploidy.” Moreover, two SNP loci were genotyped two times using different capture primer pairs (SugSNP_sh061/SugSNP_sh084 and SugSNP_sh067/SugSNP_sh092), and, as expected, the dosages of the loci diverge at higher ploidy levels (>12). These results could be explained by intrinsic problems in the molecular biology that occur during the preparation of the samples, which affects the signal intensity of the Sequenom iPLEX MassARRAY® (Sequenom Inc., San Diego, CA, USA) data.

The SuperMASSA best ploidy was equal to the genomic ploidy for six SNPs (Figure 5), and the allelic dosage confirmed in four of them. When the ploidy for the loci was fixed (8 for Region01 and 18 for Region01 and Region02 SNPs), 24 SNPs had their dosage confirmed by SuperMASSA (Figure 5—“SuperMASSA expected ploidy” columns). Notably, the estimation of the ploidy could also be a difficult task (Garcia et al., 2013), but when the ploidy used was found in BAC-FISH, the estimated dosage was in agreement with the dosage found in the BACs in 63% (28) of the SNPs (Figure 5).

**Figure 6:**
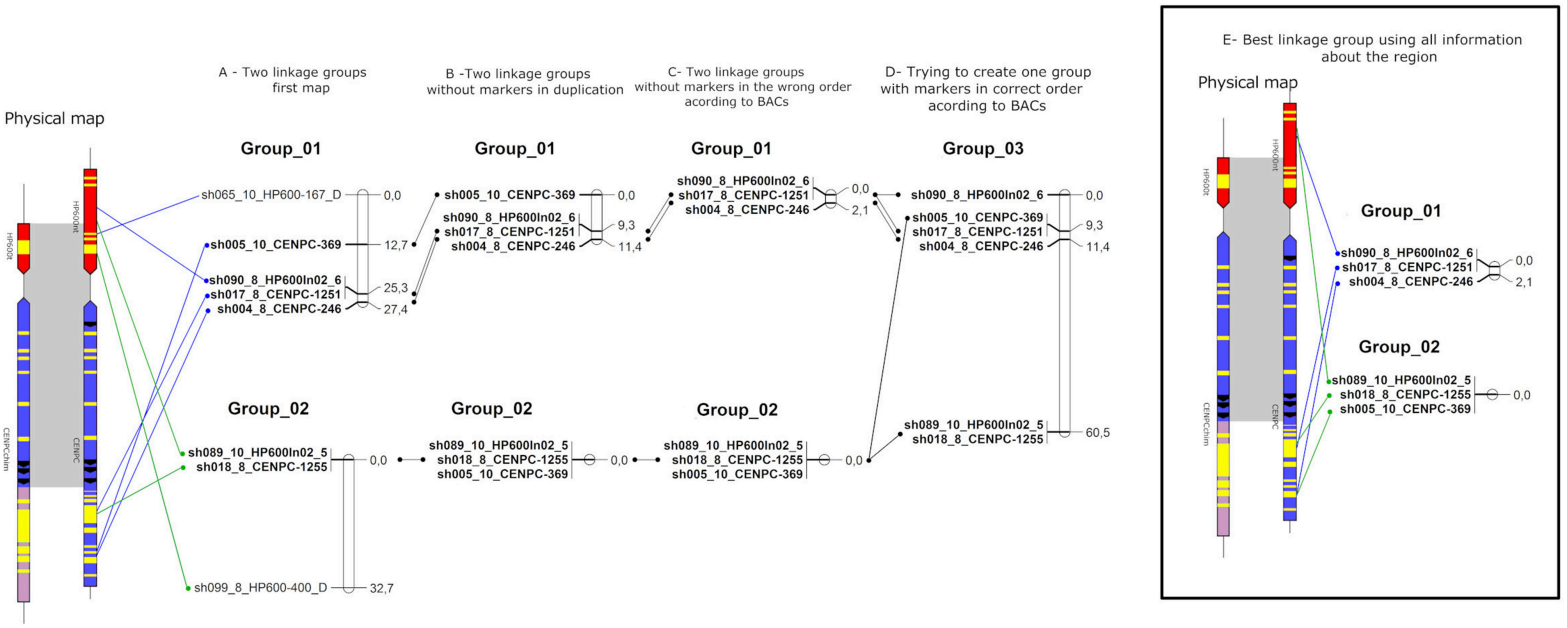
Schematic representation of the sugarcane linkage map. The sugarcane variety SP80-3280 SNPs were used to create multiple linkage maps with information about the sugarcane genome (BACs). **(A)** Linkage groups using markers in Figure 6. **(B)** Linkage groups without markers duplicated according to BACs. **(C)** Linkage groups without markers in the wrong order according to BACs. **(D)** Linkage group formed trying to create one group. **(E)** Best linkage groups using BACs information.

For the genetic mapping, 10 markers were used according to the SuperMASSA best ploidy results. First, attempts were made to add each marker to the existing linkage groups published by Balsalobre et al. (2017), but none of the markers could be linked to the groups. Then, the markers were tested for linkage among themselves. Two linkage groups could be created (Figure 6A) with 27.4 and 32.7 cM. The SugSNP_sh065 and SugSNP_sh099 markers were physically located in Region01 and Region02, respectively. It was unexpected that duplicated markers were linked to a linkage group, even weakly (the long distance between the markers and Supplementary Figure 8).

Using all the physical information, the duplicated markers (SugSNP_sh065 and SugSNP_sh099) were excluded (Figure 6B). Then, attempts were made to add the remaining markers to the groups again, and the SugSNP_sh005 marker was inserted into Linkage group 02 (Figure 6C). The markers that were in the wrong positions according to the physical map (BACs) were also excluded, and the SugSNP_sh005 marker was excluded from Linkage group 01 but remained in Linkage group 02 (Figure 6C). Then, an attempt was made to form one linkage group with the remaining markers by forcing OneMap to place the markers in a single group. Again, the size of the group was too large (60.3 cM—Figure 6D). Therefore, the best representation of the region was two linkage groups, with Linkage group 01 at 2.1 cM, and Linkage group 02 at 0 cM (Figure 6E).

## Discussion

For genetic and genomic studies, information about genomic organization is very important. Here, we report the construction of two new BAC libraries for two important Brazilian cultivars, SP80-3280 and SPIAC93-3046, with a larger number of clones and higher sugarcane genome coverage than previously reported (Tomkins et al., 1999; Le Cunff et al., 2008; Figueira et al., 2012). The number of clones in a library is directly related to the number of homeologous regions that can be recovered.

The genomic SNP variation in sugarcane coding regions has been estimated to be one SNP every 50 bp (Cordeiro et al., 2006) and one every 86 bp (Cardoso-Silva et al., 2014). For coding Region01, one SNP was found per 70 bases. When we compared Region01 and Region02, one SNP was found per 12 bases using only the data for the SP80-3280 sugarcane variety. These results revealed a high level of diversity in sugarcane, i.e., a high number of SNPs in each region, which could be used to generate molecular markers and to improve genetic maps. Moreover, the diversity rate of both regions together could be used as an indicator of a duplicated gene, i.e., a rate < 20 (see Supplementary Table 4). However, different ratios of SNPs occur across the genome (Feltus et al., 2004).

The hypothetical gene *HP600* and the *CENP-C* gene were used in this work as a case study. The function of *HP600* is unknown, but an ortholog of this gene is present in the genomes of rice (LOC_Os01g43060), maize (GRMZM2G114380) and sorghum (Sobic.003G221600). Sobic.003G221600 (ortholog of *HP600*) was also found in a QTL for BRIX (Murray et al., 2008; Mace and Jordan, 2011; Mancini et al., 2018). The *CENP-C* protein is a kinetochore component (Kato et al., 2013; Sandmann et al., 2017) physically located next to *HP600*. Here, we have demonstrated the existence of paralogous genes for *HP600* and *CENP-C* that are localized in two different homeologous sugarcane chromosome groups. The BAC haplotypes could be separated into two sugarcane homeologous groups as follows: (1) Region01 contained the collinearity region between sorghum and sugarcane *HP600* and *CENP-C* genes and (2) Region02 contained their paralogs.

Region01 was a recurrent case of high gene conservation and collinearity among sugarcane homeologs and the *S. bicolor* genome as reported by other authors (Jannoo et al., 2007; Garsmeur et al., 2011; de Setta et al., 2014; Vilela et al., 2017; Mancini et al., 2018). Region02 had a more complex genomic structure than that of Region01. Region02 contains parts of the *HP600* and *CENP-C* (paralogs) genes, and no synteny was found with sorghum genome. In Region02, a third partial gene (ortholog of Sobic.003G299500) was also found next to *CENP-C*, and transcriptome analysis revealed the fusion of partial *CENP-C* exons with the partial exons from the sugarcane ortholog of Sobic.003G299500 to form a chimeric gene. Region02 is a scrambled sugarcane sequence that was possibly formed from different noncollinear ancestral sequences, but the exonic structure of the genes was retained. Multiple events may have resulted in Region02, but the number and types (TE, translocations) of events could not be determined with our data.

No LTR retrotransposons were shared among the haplotypes from Region01 and Region02, suggesting that all LTR retrotransposon insertions occurred after the duplication. The oldest LTR retrotransposon insertions in Region02 were dated from 2.3 Mya, representing a possible age for this duplication. The presence of a set of sugarcane homeologs with very similar gene structures leads us to speculate that an ancestral event occurred prior to polyploidization (Daniels and Roach, 1987; Paterson et al., 2013) and that nobilization (Bremer, 1961) resulted in this structure.

The phylogenetic analysis of gene haplotypes from *HP600* and *CENP-C* provided evidence that the multiple genes found in maize are the result of specific duplications in the maize taxa. Given the gene organization among the BACs, sorghum and rice revealed that *HP600* and *CENP-C* were side by side, and the expected orthologs from maize could be GRMZM2G114380 (*HP600*) and GRMZM2G114315 (*CENP-C*) because only these two genes are physically side by side. The other maize orthologs were probably maize paralogs that resulted from specific duplications of the *Z. mays* genome.

The chromosome number determination of five Brazilian varieties (including SP80-3280) showed an equal number of chromosomes (2n = 112). A number of differences in the CMA/DAPI patterns were found among the different varieties analyzed in this study, suggesting differences in chromosome contents, i.e., differences in homeologous arrangement. BAC-FISH hybridization was used to indicate a ploidy of eight for Region01 and 10 for Region02. The aneuploid nature of sugarcane hybrid cultivars (D'Hont, 2005; Piperidis et al., 2010) means that they contain different numbers of homeologous chromosomes. These results suggest that the sugarcane *HP600* and the *CENP-C* gene haplotypes in Region01 were duplicated in another group of homeologous chromosomes. Moreover, the numbers of BAC haplotypes found in each region are appropriate considering the BAC-FISH results, suggesting a missing haplotype for each region. Casu et al. (2012), Xue et al. (2014), and Sun and Joyce (2017) reported different methods to quantify the copy number of endogenous genes, some of which resulted in odd copy numbers. The absence of orthologs Sobic.003G221800 and Sobic.008G134700 (see Figure 1) in some BAC haplotypes suggest a possible explanation for the odd copy numbers. We were unable to determine whether this alteration resulted from a duplication or a translocation since we do not have a single haplotype that covers the entire region.

The homologous gene expression in polyploids can be affected in different ways, i.e., the homologous genes may retain their original function, one or more copies may be silenced, or the genes may diversify in function or expression (Ohno, 1970; Lynch and Force, 2000; Hegarty et al., 2006; Buggs et al., 2011). In complex polyploids, the roles of ploidy and genome composition in possible changes in gene expression are poorly understood (Shi et al., 2015). Even in diploid organisms, this task is difficult, as different interactions can affect the expression of a gene, and not all homologs are guaranteed to contribute to a function (Birchler et al., 2005). In Region02, the haplotypes of *HP600* were not found in the transcriptome dataset (Cardoso-Silva et al., 2014; Mattiello et al., 2015), but at least two haplotypes of the *CENP-C* in Region02 (chimerical gene) were expressed. The gene haplotypes of *HP600* from Region01 exhibited unbalanced expression; i.e., for some reason, the SNP ratio in the genome did not explain the transcriptome SNP ratio. These findings could mean that apart from the duplication, *HP600* might be expressed as a single-copy gene wherein only the *HP600* haplotypes in Region01 were expressed. Additionally, we could not identify the mechanisms contributing to the unbalanced expression. Therefore, the transcripts from different tissues make us speculate that some kind of tissue-specific expression could be occurring.

These results have several implications for the integration of the transcriptome and genomic data. First, for example, a gene such as *HP600* that demonstrates single-copy behavior in the transcriptome data and the genomic behavior of a duplicated gene can cause bias in genetic mapping. Second, a chimeric gene such as the *CENP-C* haplotypes in Region02 can result in different levels of expression of the duplicated and nonduplicated gene regions in the transcriptome data. Looking at the *CENP-C* gene, if the gene expression quantification probe recovers the nonduplicated portion of the *CENP-C* gene, it will give an expression level only for the *CENP-C* haplotypes in Region01. In contrast, as this probe quantifies the duplicated region of *CENP-C*, it will result in the quantification of *CENP-C* from both Region01 and Region02 and thus overestimate the expression of *CENP-C*. Consequently, analyses of the expression of the gene for functional studies for evaluating the balance of gene expression will be biased.

Numerous molecular mechanisms are involved in the creation of new genes, such as exon shuffling, retrotransposons and gene duplications (reviewed in Long et al., 2003). Gene fusions allow the physical coupling of functions, and their occurrence in the genome increases with the genome size (Snel et al., 2000). The CENP-C motifs described by Sandmann et al. (2017) were compared with those of *CENP-C* genes in *A. thaliana, O. sativa, Z. mays*, and *S. bicolor* (see Supplementary Figure 9). The *CENP-C* haplotypes from Region02 (chimeric gene) have the same *CENP-C* motif as that in sorghum. The *CENP-C* haplotypes from Region01 have one variation in the second residue of the *CENP-C* motif: a glycine in sorghum and a valine in *CENP-C* haplotypes from Region01. This result suggests that the *CENP-C* haplotypes from Region01 and Region02 are able to bind to cenH3 nucleosomes.

When we compared *HP600* and *CENP-C* found in SP80-3280 BACs with the *S. spontaneum* genome (Zhang et al., 2018b), we confirmed (i) the presence of the duplication region found in Region02 in one chromosome allele (Chr02D); (ii) the existence of a chimeric gene formed by *CENP-C* and Sobic.003G299500 located in two alleles (Chr02D and Chr02A); and (iii) evidence that the duplication found in Region02 occurred after the separation of sorghum and before the formation of the *Saccharum* genus.

Molecular markers were also used to compare the ploidy found in BACs with the results from the SuperMASSA software (Garcia et al., 2013). SuperMASSA uses segregation ratios to estimate ploidy, which is not the same as estimating ploidy by chromosome counting because of the differences in the estimation and the real ploidy visualized. The SNPs present in a duplication were mapped in linkage groups (Figure 6A) and demonstrated a high distance between the markers in the linkage map. The size of a genetic map is a function of the recombination fraction, with the following two factors influencing the map size: (I) the number of recombinations and linkage phase found between two markers; (II) genotyping errors. In this case, the mapping of duplicated markers is an error and is interpreted by OneMap in a recombination fraction, which inflates the map.

Two markers classified with a ploidy of 10 and one with a ploidy of 8 formed Linkage group 02 (Figure 6E). The ploidy is not a determinant for the OneMap construction of a linkage group, but the recombination fraction is. In other words, recombination fractions can still be computed between single-dose markers classified in different ploidy levels. In fact, most nulliplex, simplex and duplex individuals will have the same dosage call using either 8 or 10 as the ploidy level. Additionally, the genome data (BACs and BAC-FISH) demonstrated that all markers had the same ploidy of eight and that the physical distances among the markers were too small and thus probably resulted in the lack of recombination. The fact that we obtained two linkage groups can be explained by the possibility that single-dose markers may be linked in repulsion, and insufficient information is available to assemble all of the markers into one group. Trying to calculate the recombination fraction between markers D1 and D2 (according to the nomenclature of Wu et al., 2002) in diploids presents the same obstacle. Indeed, it is a small region where the markers should segregate together, but this segregation was not observed (Figure 6A). We reported that some duplicated markers can be mapped in the linkage map. In data with no information about physical structure, the same phenomenon could occur. Then, the information about the physical structure was used to correct this bias (Figures 6B–D), forming linkage groups with markers segregating together (Figure 6E).

Once established, the polyploidy might now fuel evolution by virtue of its polyploid-specific advantages. Vegetative propagation can lead to the retention of genes (Freeling, 2017). Vegetative propagation is widely used to propagate sugarcane (even for non-domesticated sugarcanes) and could explain the high variation in sugarcane (number of SNPs located) and the high level of gene retention. The combination of divergent genomes within a hybrid can lead to immediate, profound and highly varied genome modifications, which could include chromosomal and molecular structural modifications (Shen et al., 2005; Doyle et al., 2008; Soltis and Soltis, 2009; Jiang et al., 2011) as well as epigenetic changes (Chen et al., 2010) and global transcriptomic changes (Hegarty et al., 2006; Buggs et al., 2011). The integration of the genetic, genomic, and transcriptomic data was used to explain the interaction of the two regions in sugarcane. The *HP600* and *CENP-C* duplication described in this work occurred sometime after the separation of sugarcane and sorghum and before the polyploidization of the *Saccharum* genus. This result is supported by the following information: (I) the molecular clock time; (II) the genes are present in a homeologous group of chromosomes; (III) the *CENP-k* motifs from the *CENP-C* haplotypes in Region02 are more similar to sorghum than to its paralog in sugarcane; and (IV) the duplication was observed in the *S. spontaneum* genome (Zhang et al., 2018b).

Genetic mapping remains a successful method to improve the production of crop plants. Sugarcane represents one of the crops with difficulties for producing accretive genetic maps, and this impacts the improvement of breeding programs. The variation in ploidy level among the loci and the duplicated genes play a special role in this problem. We used different approaches to show molecular events that affect the genetic mapping as well as the problems associated with defining the ploidy level and dosage among its alleles. Future attention should be given to the relationship between transcription and genomics, as exemplified by the *HP600* gene, which has a single-copy gene behavior in the transcriptome but shows a duplicated region in the genome. The genetic, genomic, and transcriptome interactions among sugarcane homeologs remain obscure. Several works have attempted to understand these interactions (Jannoo et al., 2007; Wang et al., 2010; Garsmeur et al., 2011; Casu et al., 2012; Figueira et al., 2012; Garcia et al., 2013; de Setta et al., 2014; Xue et al., 2014; Sun and Joyce, 2017; Vilela et al., 2017; Mancini et al., 2018). The high polyploidy in sugarcane cultivars makes the detection of the ploidy of a locus a challenge (Casu et al., 2012; Garcia et al., 2013; Xue et al., 2014; Sun and Joyce, 2017).

Particular emphasis should be given to the determination studies of the ploidy level and of the duplication loci with the intention of better understanding complex polyploids. These studies remain the most original and challenging in terms of understanding the sugarcane genome. This study sheds light on the influence of the genome arrangement on transcriptome and genetic map analyses in the sugarcane polyploid genome. The integration of genomic sequence arrangements, transcription profiles, cytogenetic organization and the genetic mapping approach might help to elucidate the behavior of gene expression, the genetic structure and successful sequence assembly of the sugarcane genome. Future integrated studies will undoubtedly help to enhance our understanding of complex polyploid genomes including the sugarcane genome.

## Author Contributions

AdS, DS, EF-M, HB, MV, and AG designed the study. AB, DS, HH, JF, MC, MM, MR, ND, NR, and SV performed the research. CC-S, DS, GP, MV, M-AV, and RV contributed new analytical or computational tools. AG, AB, AdS, CC-S, DS, GP, HB, LP, MM, ML, MSC, MV, and SV analyzed the data. DS, MV, and AdS wrote the paper. All authors critically read the text and approved the manuscript.

### Conflict of Interest Statement

The authors declare that the research was conducted in the absence of any commercial or financial relationships that could be construed as a potential conflict of interest.
