# Supplementary material for: Gene Duplication in the Sugarcane Genome: A Case Study of Allele Interactions and Evolutionary Patterns in Two Genic Regions
Source: Front Plant Sci. 2019 May 7;10:553. doi: 10.3389/fpls.2019.00553 (PMC6514446; doi:10.3389/fpls.2019.00553)
Supplement: Supplementary file 1 [file Data_Sheet_1.docx]

Supplementary Material

# Supplementary Data

## BAC Library Construction and BAC-End Analyses

The high-molecular-weight (HMW) DNA was prepared from the leaves as described by Peterson et al. (2000) with modifications as described by Gonthier et al. (2010). The HMW DNA was embedded in low-melt agarose (Lonza InCert™ Agarose, Lonza Rockland Inc., Rockland, ME, USA) and partially digested with HindIII (New England Biolabs, Ipswich, MA, USA). Next, two size-selection steps were performed by pulsed field gel electrophoresis (PFGE) with a Bio-Rad CHEF Mapper system (Bio-Rad Laboratories, Hercules, CA, USA), and the selected DNA was ligated into the pIndigoBAC-5 HindIII-Cloning Ready vector (Epicenter Biotechnologies, Madison, WI, USA) as described by Chalhoub et al. (2004). The insert size was verified by preparing DNA BACs with the NucleoSpin® 96 Plasmid Core Kit (MACHEREY-NAGEL GmbH & Co., Düren, Germany) according to the kit instructions, and the DNA was digested by the NotI (New England Biolabs, Ipswich, MA, USA) restriction enzyme and analyzed by PFGE.

The BAC library from the SP80-3280 sugarcane variety resulted in 221,184 clones arrayed in 576 384-well microtiter plates with a mean insert size of 110 kb. This BAC library was approximately 2.4 genome equivalents (10 Gb) and 26 monoploid genome equivalents (930 Mb, Figueira et al., 2012). For the IACSP93-3046 sugarcane variety, the library construction resulted in 165,888 clones arrayed in 432 384-well microtiter plates with a mean insert size of 110 kb, which is approximately 1.8 genome equivalents and 19 monoploid genome equivalents.

For the BAC-end sequencing (BES), 384 random BAC DNAs from each library were prepared with the NucleoSpin® 96 Plasmid Core Kit (MACHEREY-NAGEL GmbH & Co., Düren, Germany) according to the kit instructions. The sequencing reactions were performed according to the manufacturer's instructions for the BigDye Terminator Kit (Applied Biosystems, Foster City, CA, USA). The primers used in the reactions were T7 Forward (5’ TAATACGACTCACTATAGG 3’) and M13 Reverse (5’ AACAGCTATGACCATG 3’). The PCR conditions were 95°C for 1 min followed by 90 cycles of 20 sec at 95°C, 20 sec at 50°C and 4 min at 60°C. The samples were loaded on a 3730xl DNA Analyzer (Applied Biosystems). Sequence trimming was conducted by processing the traces using the base-calling software PHRED (Ewing and Green, 1998; Ewing et al., 1998), and reads with a phred score < 20 were trimmed. The sequences were compared using BLASTN with the *S. bicolor* genome from Phytozome v10.1 (Goodstein et al., 2012). Only clones with forward and reverse sequence maps in the *S. bicolor* genome, a maximum distance of 600 kb and no hits with repetitive elements were used to anchor the *S. bicolor* genome.

BES resulted in an overview of the genome and validated the clones obtained through library construction. The SP80-3280 BAC library yielded 650 (84.6%) good BES sequences, of which 319 sequences had repetitive elements and 92 exhibited similarities with sorghum genes. Excluding hits for more than one gene (probably duplicated genes or family genes), 65 sequences could be mapped to the *S. bicolor* genome (see Supplementary Figure 1, Supplementary Material). The BAC library for IACSP93-3046 yielded 723 (94%) good BES sequences, of which 368 sequences exhibited the presence of repetitive sequences and 111 exhibited similarity with at least one gene. Excluding genes with hits for more than one gene, 74 of the sequences could be mapped to the *S. bicolor* genome (see Supplementary Figure 1, Supplementary Material).

The approach of mapping the BES in the *S. bicolor* genome, already performed for other libraries (Figueira et al., 2012; Kim et al., 2013; Visendi et al., 2016), revealed high synteny with the *S. bicolor* genome and a large number of TEs in the sugarcane genome. Kim et al. (2013) showed BES anchorage of approximately 6.4%, and Figueira et al. (2012) showed anchorage of approximately 22%. Our data showed 10% BES anchorage in the sorghum genome for both libraries constructed. These results are more similar to those of Kim et al. (2013), since they used only BES ≥ 300 bp and we used BES ≥100 bp.

The sugarcane genome has been reported to be composed of approximately 40% TEs (Figueira et al., 2012; Kim et al., 2013; de Setta et al., 2014). We also found that the average percentage of TEs was 40%, but this value has a very large variance among the haplotypes, with a minimum of 21% and a maximum of 65%. Figueira et al. (2012) and De Setta et al. (2014) also revealed an inflation in the sugarcane genome in comparison with the *S. bicolor* genome. De Setta et al. (2014) reported a very significant expansion that mainly occurred in the intergenic and intronic regions and was primarily because of the presence of TE, and we confirmed this report by comparing our data with data on the *S. bicolor* genome. Several studies have reported a very significant sugarcane genome expansion (Jannoo et al., 2007; Wang et al., 2010; Garsmeur et al., 2011; Figueira et al., 2012; de Setta et al., 2014; Vilela et al., 2017; Mancini et al., 2018).

## Target Gene Determination, BAC Library Screening and BAC Annotation

*S. bicolor*, *Z. mays* and *O. sativa* transcripts were obtained from Phytozome v10.1 (Goodstein et al., 2012). Each transcript was queried against itself, and orthologous genes that resulted in redundant sequences were eliminated. From the remaining genes, the gene Sobic.003G221600 (*Sorghum bicolor* v3.1.1 – Phytozome v. 12) was chosen because it was inserted in a QTL for Brix from a study by Murray et al. (2008), which identified the QTL in the SB-03 genome (*S. bicolor* v3.1.1 – Phytozome v. 12). The sequence of the gene Sobic.003G221600 was then used as a query in the SUCEST-FUN database (http://sucest-fun.org/ - Vettore et al., 2003) and the transcriptome obtained by Cardoso-Silva et al. (2014) to recover sugarcane transcripts. All the obtained transcripts were aligned (MAFFT; (Katoh et al., 2002)) to generate phylogenetic trees using the maximum likelihood method (PhylML 3.0; (Guindon and Gascuel, 2003)). A search in OrthoDB database also demonstrated the gene Sobic.003G221600 was in a single copy in the diploid grass group (OrthoDB, Kriventseva et al., 2018).

The sugarcane transcripts were split into exons according to their annotation in *S. bicolor*, *Z. mays* and *O. sativa,* and exon five was used to design the probe to screen both BAC libraries (F: 5’ ATCTGCTTCTTGGTGTTGCTG 3’, R: 5’ GTCAGACACGATAGGTTTGTC 3’). DNA fragments were PCR-amplified from sugarcane SP80-3280 and SPIAC93-3046 genomic DNA with specific primers targeting the Sobic.003G221600 gene. The PCR amplification conditions were 95°C for 8 min; 30 cycles of 20 sec denaturation at 95°C, 20 sec of annealing at 60°C, and a 40 sec extension at 72°C; and a final 10 min extension at 72°C. The probes were sequenced before screening the BAC library.

Both BAC libraries were spotted onto high-density colony filters with the QPix2 XT workstation (Molecular Devices, Sunnyvale, CA, USA). The BAC clones were spotted in duplicate using a 7 × 7 pattern onto 22 × 22 cm Immobilon-Ny+ filters (Molecular Devices). The whole BAC library from the SP80-3280 sugarcane variety was spotted on four sets of filters, each with 55,296 clones in duplicate, and the whole BAC library from SPIAC93-3046 sugarcane variety was spotted on three sets of filters, each with 55,296 clones in duplicate. The filters were processed as described by Roselli et al. (2017). Probe radiolabeling and filter hybridization were performed as described in Gonthier et al. (2010).

The SP80-3280 BAC library was used to construct a 3D pool. A total of 110,592 clones were pooled into 12 superpools following the protocol used by Paux et al. (2008). The positive BAC clones from the SP80-3280 library were isolated, and one isolated clone was validated by qPCR. The insert size of each BAC was estimated by using an electrophoretic profile of NotI-digested BAC DNA fragments and observed by PFGE (CHEF-DRIII system, Bio-Rad) in a 1% agarose gel in 0.5× TBE buffer under the conditions described in Paiva et al. (2011).

Twenty-two positive BAC clones were sequenced in pools of 10 clones. One microgram of each BAC clone was used to prepare individual tagged libraries with the GS FLX Titanium Rapid Library Preparation Kit (Roche, Branford, CT, USA). BAC inserts were sequenced by pyrosequencing with a Roche GS FLX Life Sciences instrument (Branford, CT, USA) in CNRGV, Toulouse, France.

The sequences were trimmed with PHRED, vector pIndigoBAC-5 sequences and the *Escherichia coli* str. K12 substr. DH10B complete genome were masked using CROSS_MATCH, and the sequences were assembled with PHRAP (Gordon et al., 1998; Gordon et al., 2001; Gordon, 2003) as described by De Setta et al. (2014). A BLASTN with the draft genome (Riaño-Pachón and Mattiello, 2017) was performed. A search was performed in the NCBI databank to find sugarcane BACs that could possibly have the target gene *HP600*.

The *HP600* gene was used as a target gene and showed strong evidence of being a single-copy gene when the *HP600* transcripts from sorghum, rice and sugarcane were compared. Twenty-two BAC clones from the SP80-3280 library that had the *HP600* target gene (NCBI from MH463467 to MH463488) and a previously sequenced BAC (Mancini et al. (2018); NCBI Accession Number MF737011) were sequenced by Roche 454 sequencing (see Supplementary Table 1, Supplementary Material). The BACs varied in size from 48 kb (Shy171E23) to 162 kb (Shy432H18), with a mean size of 109 kb. The BACs were compared, and BACs with at least 99% similarity were considered the same haplotype (Figures 1 and 2), resulting in sixteen haplotypes. Indeed, the possibility of one homeolog being more than 99% similar to another exists, but a real haplotype cannot be distinguished from an assembly mismatch.

The BACs were first annotated with regard to the transposable elements (TEs). The TEs accounted for 21% to 65% of the sequenced bases with a mean of 40% (see Supplementary Table 1, Supplementary Material). Annotation of the TEs in the 22 BACs revealed 618 TEs (220 TEs were grouped in the same type) with sizes ranging from 97 bp to 18,194 bp.

Gene annotation (see Supplementary Tables 2 and 3, Supplementary Material) resulted in three to nine genes per BAC, with a mean of five genes per BAC (see Supplementary Table 1, Supplementary Material). The Sobic.003G221600 gene, which was used to screen the library, codes for a hypothetical protein called *HP600* in sugarcane that has been found to be expressed in sorghum and rice. A phylogenetic analysis using sorghum, rice and *Arabidopsis thaliana* transcripts revealed that this gene is probably a single-copy gene. The Sobic.003G221500 gene is a *CENP-C* ortholog in sugarcane (*S. officinarum*, haplotypes CENP-C1 and CENP-C2, described by Talbert et al. (2004)). The *HP600* and *CENP-C* sugarcane genes were found to be side by side in the sugarcane haplotypes, as in *S. bicolor* and *Oryza* *sativa* L.

## Including *Miscanthus sinensis* in Phylogenetic Analysis

In *Supplementary Figure 10,* we report the inclusion of *Miscanthus sinensis* in phylogenetic analysis. *Miscanthus sinensis* did not have the duplication (Region02) or, in *Saccharum spontaneum*, as in maze, *Miscanthus sinensis* had another CENP-C ortholog. *M. sinensis* CENP-C grouped with the CENP-C haplotypes of Region01.

# Supplementary Figures and Tables

## Supplementary Figures

**
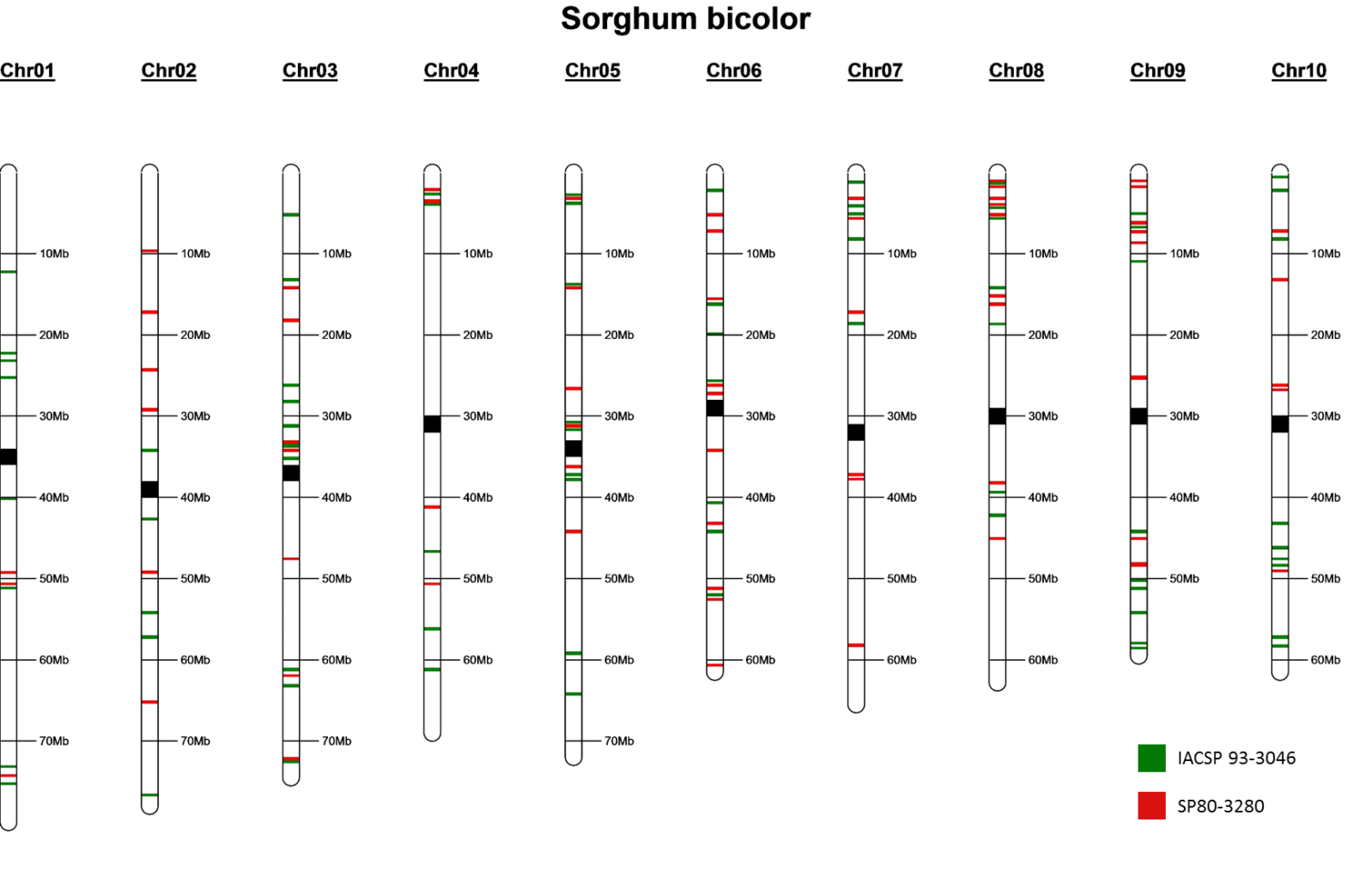
**

**Supplementary Figure 1.** BAC-end locations in the *Sorghum* genome according to BLASTn analysis. Schematic representation of the *Sorghum bicolor* genome with 10 chromosomes. The red (sugarcane variety SP80-3280) and green (sugarcane variety IACSP 93-3046) lines show the locations of the paired BAC-end sequences. Black indicates the approximate the position of the *Sorghum bicolor* centromeres.


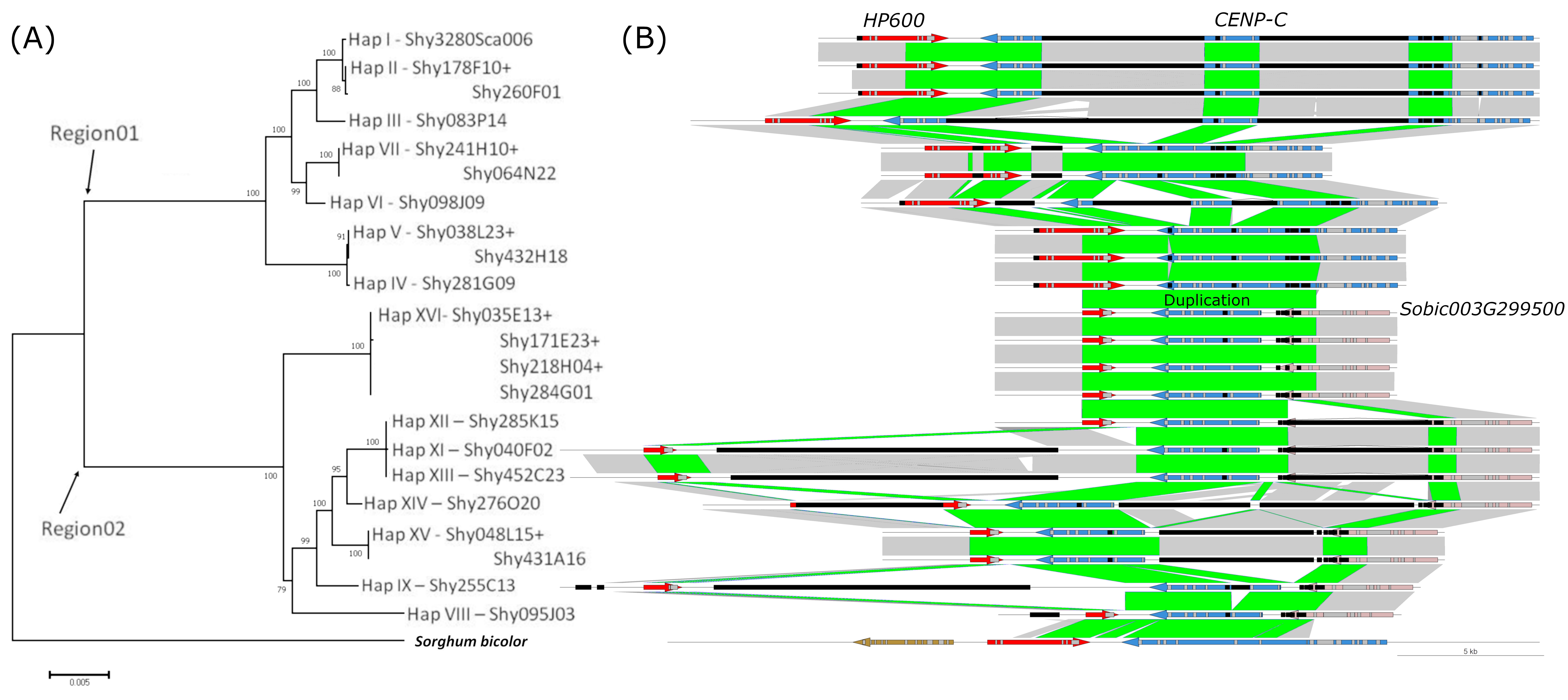


**Supplementary Figure 2.** Schematic representation of phylogenetics and physical duplications. Panel A: Evolutionary relationships between the duplications found in the 22 sugarcane BACs compared with the same region in *Sorghum bicolor*. Sugarcane BAC Shy231B24 was not included in this analysis because the BAC ends lie in the middle of the duplication. The evolutionary history was inferred using the neighbor-joining method (Saitou and Nei, 1987). The optimal tree with a total branch length = 0.12671921 is shown. The percentages of replicate trees in which the associated taxa clustered together in the bootstrap test (1000 replicates) are shown next to the branches (Felsenstein, 1985). The tree is drawn to scale, with branch lengths in the same units as the evolutionary distances used to infer the phylogenetic tree. The evolutionary distances were computed using the Kimura 2-parameter method (Kimura, 1980) and are given in units of the number of base substitutions per site. The analysis involved 23 nucleotide sequences. All positions containing gaps and missing data were eliminated. The final dataset included a total of 7025 positions. Evolutionary analyses were conducted in MEGA7 (Kumar et al., 2016). Panel B: Physical representation of the duplications from each BAC according to evolutionary relationships. Green represents the duplications; red represents the HP600 gene; and light blue represents the CENP-C gene. Light pink represents a partial ortholog of the sorghum gene Sobic003G299500. Light gray represents the relationships among BACs outside of the duplicated region. The arrows at the ends of genes HP600, CENP-C and Sobic003G299500 indicate the direction of translation.

**
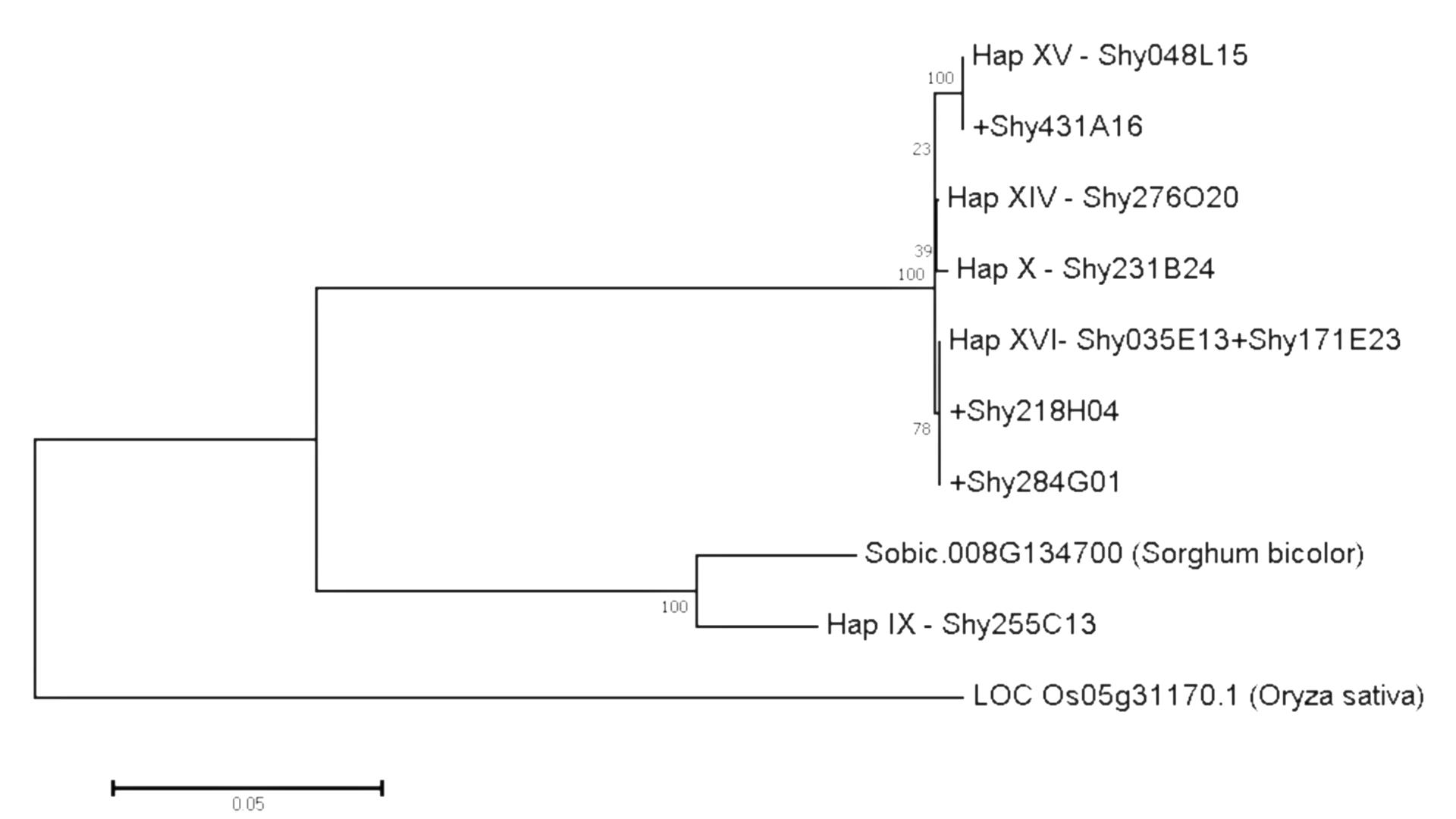
**

**Supplementary Figure 3.** Evolutionary relationships of the Sobic.008G134700 gene. The evolutionary history was inferred using the neighbor-joining method (Saitou and Nei, 1987). The optimal tree with a total branch length = 0.47067278 is shown. The percentages of replicate trees in which the associated taxa clustered together in the bootstrap test (1000 replicates) are shown next to the branches (Felsenstein, 1985). The tree is drawn to scale, with branch lengths in the same units as the evolutionary distances used to infer the phylogenetic tree. The evolutionary distances were computed using the Kimura 2-parameter method (Kimura, 1980) and are in units of the number of base substitutions per site. The analysis involved 10 nucleotide sequences. The codon positions included were the 1st+2nd+3rd+Noncoding positions. All positions containing gaps and missing data were eliminated. The final dataset included a total of 1296 positions. Evolutionary analyses were conducted in MEGA7 (Kumar et al., 2016).


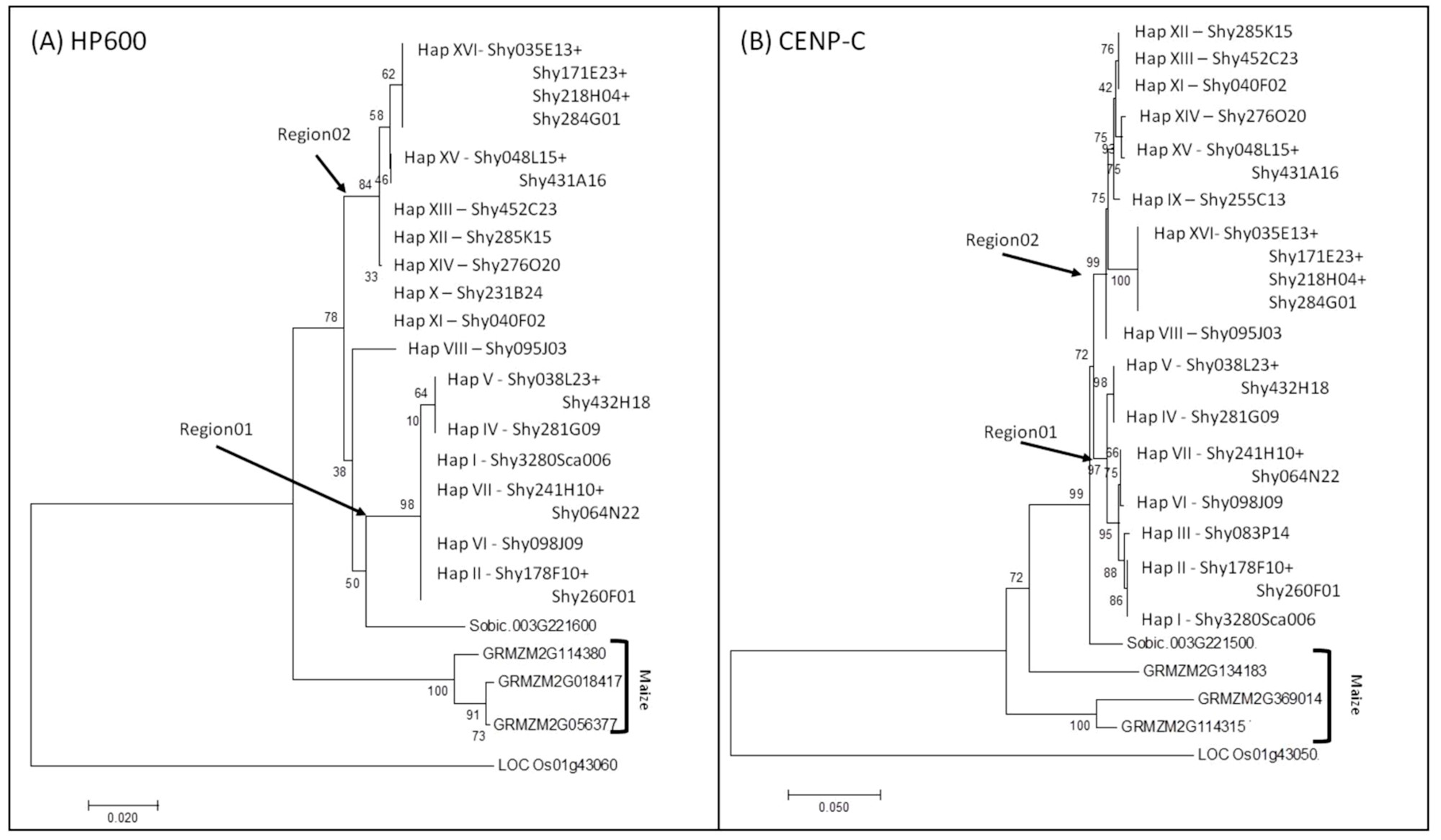


**Supplementary Figure 4.** Evolutionary relationships of HP600 and CENP-C. Panel A: HP600 evolutionary relationships among the HP600 sugarcane haplotypes in both regions in sorghum, maize (with paralogs) and rice. The haplotypes from BACs Shy083P14 and Shy255C13 were not used in the analyses because both exhibited a frame shift. There was a total of 100 positions in the final dataset. The optimal tree with a total branch length = 0.48797240 is shown. Panel B: Evolutionary relationships of the CEMP-C haplotypes in both regions in sorghum, maize (with paralogs) and rice. There was a total of 608 positions in the final dataset. The optimal tree with a total branch length = 0.70555298 is shown. The evolutionary history was inferred using the neighbor-joining method (Saitou and Nei, 1987). The percentages of replicate trees in which the associated taxa clustered together in the bootstrap test (1000 replicates) are shown next to the branches (Felsenstein, 1985). The tree is drawn to scale, with the branch lengths in the same units as those of the evolutionary distances used to infer the phylogenetic tree. Evolutionary distances were computed using the Kimura 2-parameter method (Kimura, 1980) and are in units of the number of base substitutions per site. The codon positions included were the 1st+2nd+3rd+Noncoding positions. All positions containing gaps and missing data were eliminated. Evolutionary analyses were conducted in MEGA7 (Kumar et al., 2016).

| 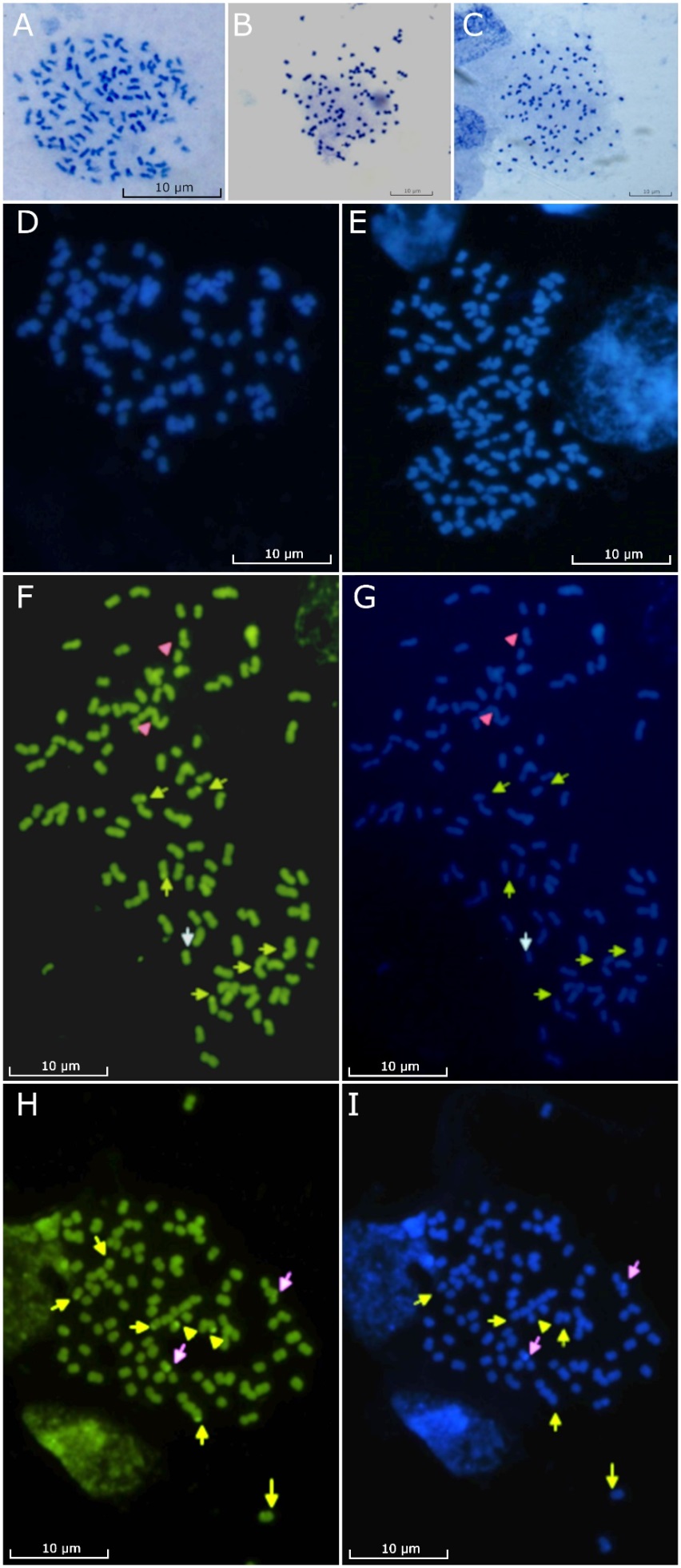 |
| --- |

**Supplementary Figure 5**. Mitotic metaphases of the sugarcane varieties. Panel A: Variety RB835486 with approximately 2n = 112 chromosomes. Giemsa staining. Panel B: Variety IACSP95-3018 with approximately 2n = 112 chromosomes. Giemsa staining. Panel C: Variety IACSP93-3046 with approximately 2n = 112 chromosomes. Giemsa staining. Panel D: SP803280 with approximately 2n = 110 chromosomes. DAPI staining. Panel E: SP81-3250 with approximately 2n = 114 chromosomes. DAPI staining. Panels F and G: CMA/DAPI banding in the sugarcane variety IACSP 93-3046. The yellow arrows indicate the six CMA^+^ (F) and DAPI^-^ (G) terminal sites. The pink arrows indicate adjacent CMA^+^ (F) and DAPI^+^ (G) sites on the same chromosome. The light blue arrow indicates a CMA^+^ (F) and DAPI^-^ (G) site. Panels H and I: CMA/DAPI banding in the sugarcane variety IACSP 95-3018. The yellow arrows indicate the seven CMA^+^ (H) and DAPI^-^ (I) terminal sites. The pink arrows indicate the chromosomes with adjacent CMA^+^ (H) and DAPI^+^ (I) sites; one site is located at the intercalary position and the other is located at the terminal position.


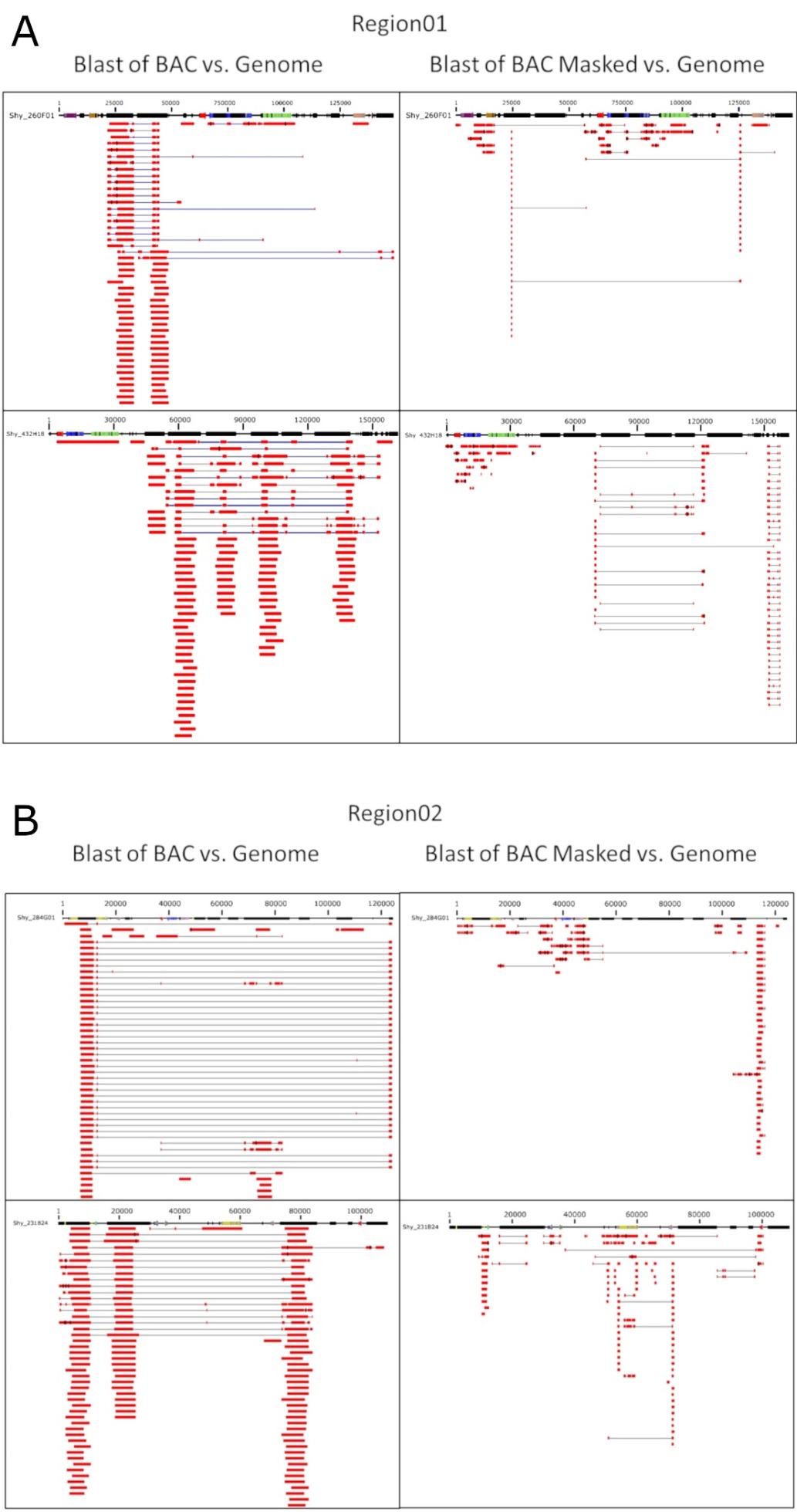


**Supplementary Figure 6.** BAC BLASTn analysis against sugarcane genome contigs. BLASTn analysis of the sugarcane genome (GCA_002018215.1 – 199.028 sequences) against sugarcane BAC clones. Panel A: BAC Shy_260F01 and BAC Shy_432H18 (Region01) BLAST results. On the left, the repeat regions are not masked. On the right, the repeat regions are masked. Panel B: BAC Shy_284G01 and BAC Shy_231B24 (Region02) BLAST results. On the left, the repeat regions are not masked. On the right, the repeat regions are masked.

**
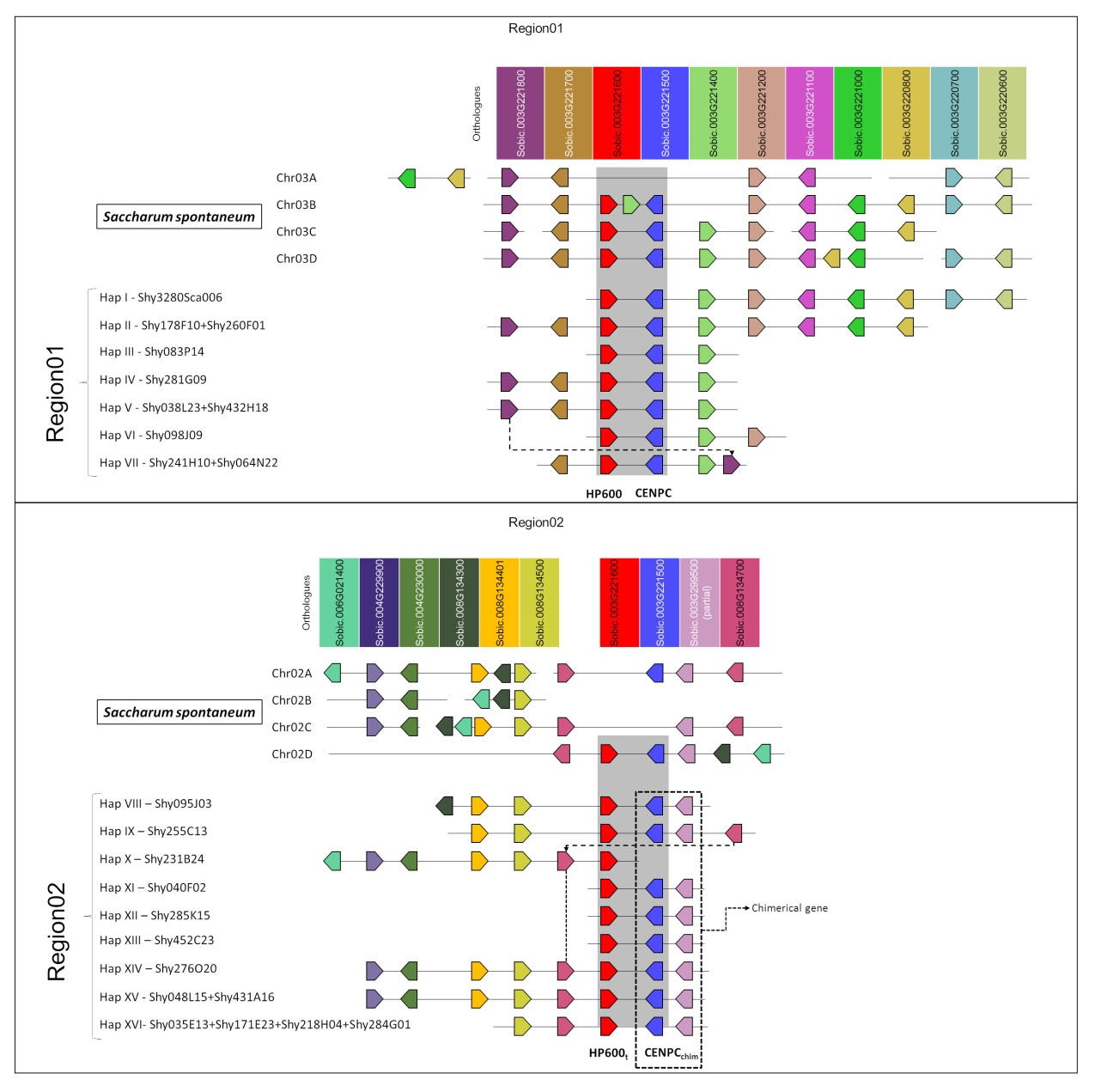
**

**Supplementary Figure 7.** Schematic comparison with the *S. spontaneum* genome. A schematic comparison between *S. spontaneum* and Region01 and Region02 BACs.

**
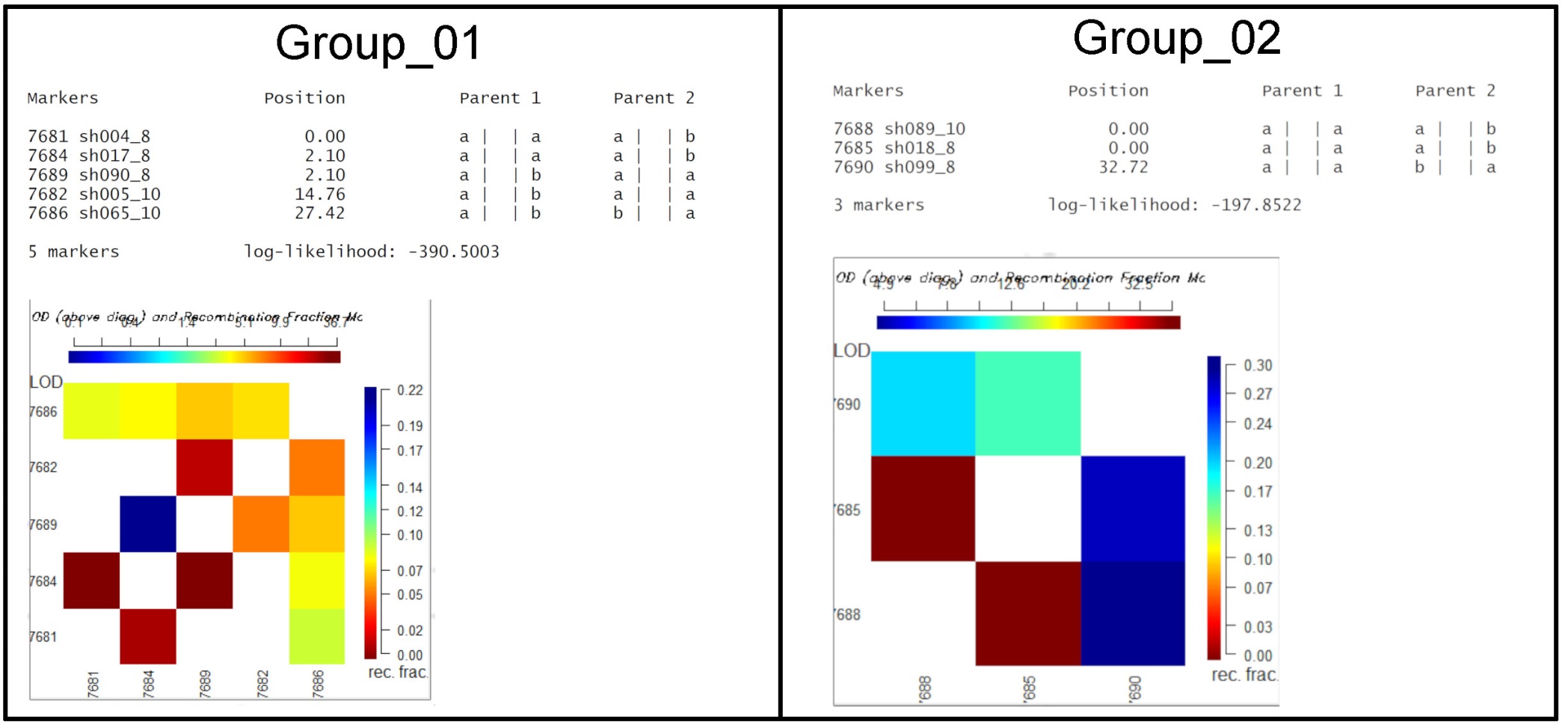
**

**Supplementary Figure 8.** Genetic map. The genetic map without the physical information. The markers SugSNP_sh065 and SugSNP_sh099 are weakly linked, but were physically located in both Region01 and Region02.

**
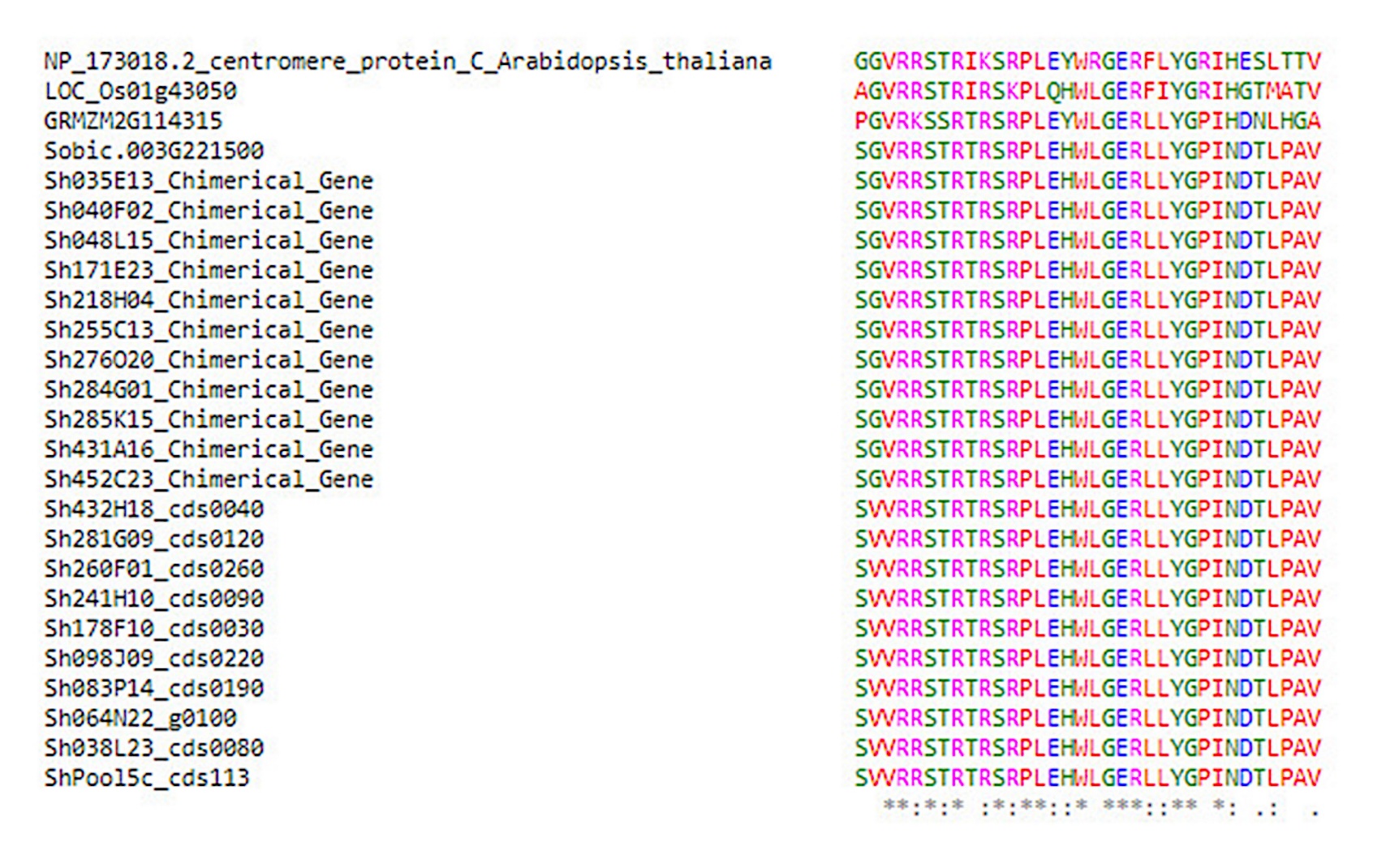
**

**Supplementary Figure 9.** CENP-C motifs alignment. Alignment of the CENP-C motifs in *Arabidopsis thaliana*, *Oryza sativa*, *Zea mays*, *Sorghum bicolor* and sugarcane BACs.

**
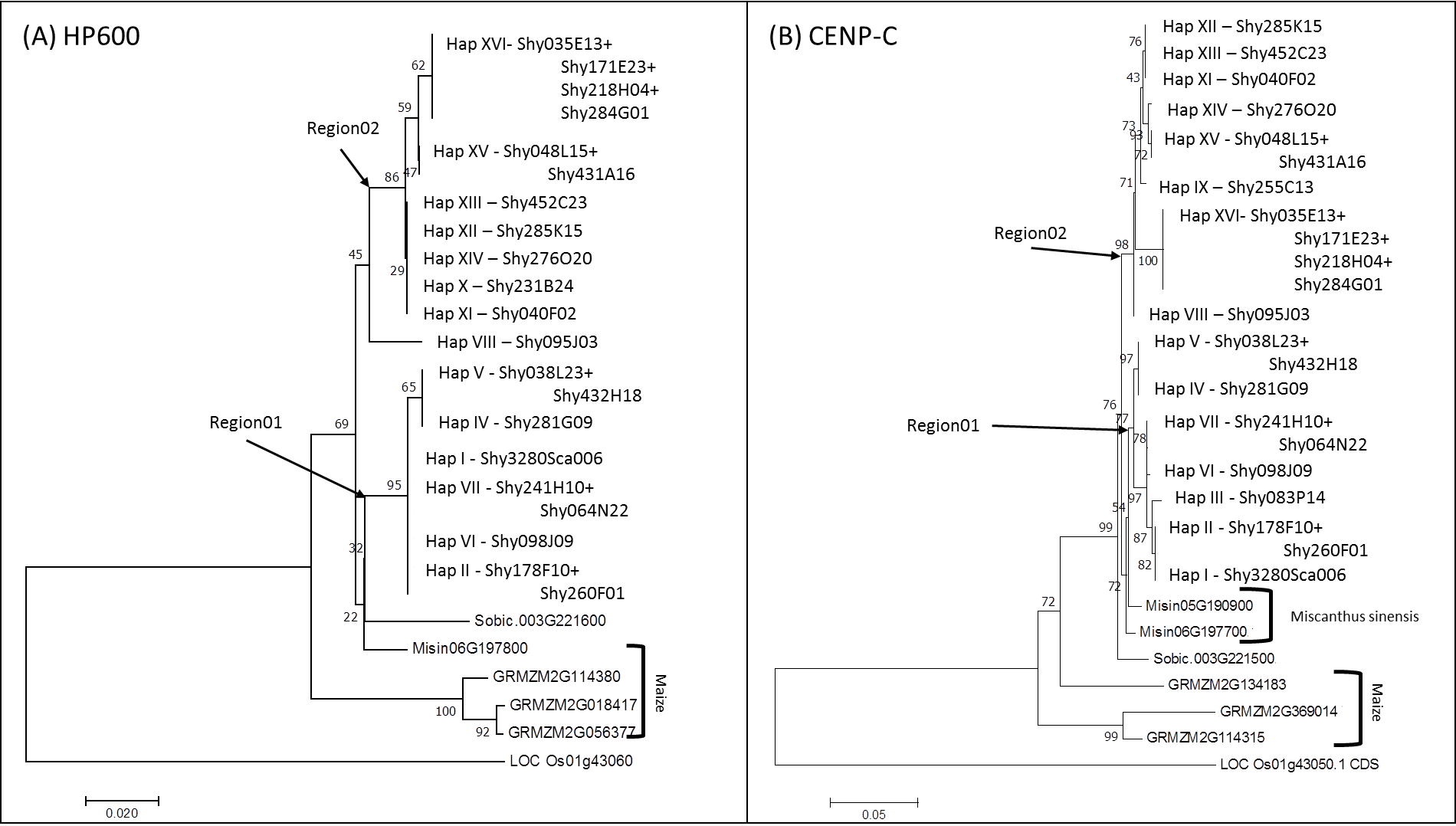
**

**Supplementary Figure 10.** Evolutionary relationships of HP600 and CENP-C including *Miscanthus sinensis*. Panel A: HP600 evolutionary relationships among the HP600 sugarcane haplotypes in both regions in sorghum, *Miscanthus sinensis*, maize (with paralogs) and rice. The haplotypes from BACs Shy083P14 and Shy255C13 were not used in the analyses because both exhibited a frame shift. There was a total of 100 positions in the final dataset. The optimal tree with a total branch length = 0.38058430 is shown. Panel B: Evolutionary relationships of the CEMP-C haplotypes in both regions in sorghum, *Miscanthus sinensis,* maize (with paralogs) and rice. There was a total of 608 positions in the final dataset. The optimal tree with a total branch length = 0.71750525 is shown. The evolutionary history was inferred using the neighbor-joining method (Saitou and Nei, 1987). The percentages of replicate trees in which the associated taxa clustered together in the bootstrap test (1000 replicates) are shown next to the branches (Felsenstein, 1985). The tree is drawn to scale, with the branch lengths in the same units as those of the evolutionary distances used to infer the phylogenetic tree. Evolutionary distances were computed using the Kimura 2-parameter method (Kimura, 1980) and are in units of the number of base substitutions per site. The codon positions included were the 1st+2nd+3rd+Noncoding positions. All positions containing gaps and missing data were eliminated. Evolutionary analyses were conducted in MEGA7 (Kumar et al., 2016).

## Supplementary Tables

**Supplementary Table 1.** BAC assembly and annotation. Summary of the assembled sugarcane BACs.

| BAC Name | Assembly | | | | | | | Region | Length w/ Gaps | Transposable Elements (TEs) | | | | Genes | Average Distance by Gene |
| --- | --- | --- | --- | --- | --- | --- | --- | --- | --- | --- | --- | --- | --- | --- | --- |
|  | Sequencing Technology | Reads | Read Mean Size | Total Bases | Length w/o Gaps | Coverage | Gaps |  |  | Annotated | | Predicted | |  |  |
|  |  |  |  |  |  |  |  |  |  | Bases | % | Bases | % |  |  |
| Shy038L23 | Roche 454 | 17,577 | 406 | 7,131,876 | 84,182 | 85 | 1 | Region01 | 84,282 | 23,814 | 28% | 37,309 | 44% | 5 | 16,856 |
| Shy064N22 | Roche 454 | 10,412 | 413 | 4,297,624 | 91,701 | 47 | 1 | Region01 | 91,801 | 20,718 | 23% | 40,179 | 44% | 4 | 22,950 |
| Shy083P14 | Roche 454 | 4,877 | 394 | 1,920,945 | 99,905 | 19 | 1 | Region01 | 100,005 | 35,056 | 35% | 53,290 | 53% | 3 | 33,335 |
| Shy098J09 | Roche 454 | 6,157 | 402 | 2,474,363 | 98,874 | 25 | 1 | Region01 | 98,974 | 26,863 | 27% | 35,883 | 36% | 4 | 24,744 |
| Shy178F10 | Roche 454 | 15,961 | 445 | 7,104,720 | 111,364 | 64 | - | Region01 | 111,364 | 59,807 | 54% | 50,537 | 45% | 7 | 15,909 |
| Shy241H10 | Roche 454 | 31,589 | 290 | 9,146,219 | 134,894 | 68 | 1 | Region01 | 134,994 | 40,990 | 30% | 66,474 | 49% | 5 | 26,999 |
| Shy260F01 | Roche 454 | 4,346 | 450 | 1,954,785 | 148,093 | 13 | - | Region01 | 148,093 | 69,918 | 47% | 74,438 | 50% | 7 | 21,156 |
| Shy281G09 | Roche 454 | 34,579 | 298 | 10,298,703 | 130,914 | 79 | - | Region01 | 130,914 | 47,495 | 36% | 69,302 | 53% | 5 | 26,183 |
| Shy432H18 | Roche 454 | 15,860 | 424 | 6,727,971 | 162,512 | 41 | - | Region01 | 162,512 | 104,933 | 65% | 108,974 | 67% | 3 | 54,171 |
| Shy3280Sca006 | PacBio | 60,844 | 1,274 | 77,515,256 | 135,057 | 574 | - | Region01 | 135,057 | 44,047 | 33% | 55,205 | 41% | 9 | 15,006 |
| Shy035E13 | Roche 454 | 6,188 | 398 | 2,462,177 | 105,606 | 23 | 1 | Region02 | 105,706 | 43,471 | 41% | 54,550 | 52% | 5 | 21,141 |
| Shy040F02 | Roche 454 | 7,402 | 399 | 2,955,331 | 89,075 | 33 | 1 | Region02 | 89,175 | 48,587 | 54% | 61,155 | 69% | 3 | 29,725 |
| Shy048L15 | Roche 454 | 4,909 | 384 | 1,884,792 | 83,194 | 23 | 1 | Region02 | 83,294 | 29,467 | 35% | 37,594 | 45% | 6 | 16,659 |
| Shy095J03 | Roche 454 | 5,315 | 412 | 2,190,264 | 90,786 | 24 | - | Region02 | 90,786 | 36,285 | 40% | 43,174 | 48% | 6 | 18,157 |
| Shy171E23 | Roche 454 | 6,349 | 448 | 2,845,515 | 48,796 | 58 | 1 | Region02 | 48,896 | 27,884 | 57% | 27,875 | 57% | 3 | 16,299 |
| Shy218H04 | Roche 454 | 20,668 | 299 | 6,172,319 | 68,037 | 91 | 1 | Region02 | 68,137 | 14,042 | 21% | 28,951 | 42% | 5 | 13,627 |
| Shy231B24 | Roche 454 | 22,282 | 296 | 6,588,557 | 107,057 | 62 | 2 | Region02 | 107,257 | 48,126 | 45% | 64,756 | 60% | 7 | 17,876 |
| Shy255C13 | Roche 454 | 15,045 | 440 | 6,615,510 | 151,415 | 44 | 2 | Region02 | 151,615 | 59,481 | 39% | 73,712 | 49% | 6 | 30,323 |
| Shy276O20 | Roche 454 | 16,919 | 443 | 7,490,042 | 105,869 | 71 | - | Region02 | 105,869 | 45,134 | 43% | 64,911 | 61% | 8 | 15,124 |
| Shy284G01 | Roche 454 | 12,607 | 447 | 5,630,527 | 122,961 | 46 | 1 | Region02 | 123,061 | 51,904 | 42% | 56,501 | 46% | 5 | 24,612 |
| Shy285K15 | Roche 454 | 14,393 | 436 | 6,273,629 | 99,815 | 63 | 3 | Region02 | 100,115 | 37,220 | 37% | 58,277 | 58% | 3 | 33,372 |
| Shy431A16 | Roche 454 | 10,993 | 392 | 4,308,085 | 132,490 | 33 | 1 | Region02 | 132,590 | 58,443 | 44% | 70,437 | 53% | 8 | 18,941 |
| Shy452C23 | Roche 454 | 16,361 | 283 | 4,637,892 | 100,514 | 46 | 2 | 2 | 100,714 | 53,185 | 53% | 61,428 | 61% | 3 | 33,571 |
| Mean | | 15,723 | 429 | 8,201,178 | 108,831 | 71 |  |  | 108,922 | 44,647 | 40% | 56,301 | 51% | 5 | 23,771 |

**Supplementary Table 2.** Orthologous genes from Region01. Region01 orthologous genes found in sugarcane BACs. “+” indicates that the chromosome has partial genes and “*” indicates more than one copy.

| Sorghum | Gene | BAC Genes | Sucest-Fun | S. Spontaneum [Zhang et al., 2018] | Rice | Maize |
| --- | --- | --- | --- | --- | --- | --- |
| Sobic.003G221800 | Putative uncharacterized protein (C5XR27) - Probable aldo-keto reductase 5 | Sh241H10_g0150, Sh260F01_g0020, Sh281G09_g0030 | SCMCLV1032B11 | Chr3A, Chr3B* and +, Chr3C* and +, Chr3D* and +, | LOC_Os01g43090 | GRMZM2G024315 |
| Sobic.003G221700 | Putative uncharacterized protein (B6U3Y6) – Similar to calcium ion binding related to photosystems II | Sh038L23_g0290, Sh064N22_g0030, Sh241H10_g0030, Sh260F01_g0060, Sh281G09_g0060 | SCQSAM1030F07 | Chr3A, Chr3B, Chr3C, Chr3D | LOC_Os01g43070 | GRMZM2G000256 |
| Sobic.003G221600 | Putative uncharacterized protein (C5XR26) | Sh038L23_g0100, ShPool5c_g118, Sh064N22_g0080, Sh083P14_g0220, Sh098J09_g0230, Sh178F10_g0010, Sh241H10_g0060, Sh260F01_g0210, Sh281G09_g0090, Sh432H18_g0030 | SCCCSB1004A04 | Chr2D+, Chr3B, Chr3C, Chr3D* and + | LOC_Os01g43060 | GRMZM2G114380 |
| Sobic.003G221500 | CENP-C1 (Q66LH0) - CENTROMERE PROTEIN C | Sh064N22_g0100, Sh083P14_g0190, ShPool5c_g113, Sh098J09_g0220, Sh178F10_g0030, Sh241H10_g0090, Sh260F01_g0260 | SCSGFL4190C08 | Chr2A+, Chr2D+,  Chr3B, Chr3C, Chr3D* and +,  Chr7B+ | LOC_Os01g43050 | GRMZM2G114315 |
|  | CENP-C2 (Q66LH1) - CENTROMERE PROTEIN C | Sh038L23_g0080, Sh281G09_g0120, Sh432H18_g0040 |  |  |  |  |
| Sobic.003G221400 | Putative uncharacterized protein (C5XR25) - Similar to acetyltransferase 1-like | Sh038L23_g0060, Sh064N22_g0120, Sh083P14_g0160, ShPool5c_g090, Sh098J09_g0140, Sh178F10_g0080, Sh241H10_g0120, Sh260F01_g0280, Sh281G09_g0150, Sh432H18_g0080 | SCJFRZ2031F10 | Chr2D+,  Chr3B, Chr3C, Chr3D* | LOC_Os01g43030 | GRMZM2G047093 |
| Sobic.003G221200 | CTP synthase (C5XR23) | Sh178F10_g0170, ShPool5c_g051, Sh260F01_g0360 | SCCCAM1C03H03 | Chr3A* and +, Chr3B+, Chr3C, Chr3D | LOC_Os01g43020 | GRMZM2G153058 |
| Sobic.003G221100 | Putative uncharacterized protein (C5XR21) | Sh178F10_g0190, ShPool5c_g043, Sh260F01_g0380 | SCSFFL8044H03 | Chr3A*, Chr3B, Chr3C, Chr3D | LOC_Os02g37140 | AC209364.3_FGP009 |
| Sobic.003G221000 | Putative uncharacterized protein (C5XR20) - Similar to membrane-associated salt-inducible protein-like | Sh178F10_g0260, Shpool5c_g028 | SCEZHR1054H09 | Chr3A, Chr3B, Chr3C, Chr3D | LOC_Os01g42990 | GRMZM2G369931 |
| Sobic.003G220800 | Putative uncharacterized protein (C5XR18) - RNA recognition motif (RRM)-containing protein-like | ShPool5c_g022, Sh178F10_g0290 | No hit | Chr3A, Chr3B, Chr3C, Chr3D | LOC_Os08g23120 | GRMZM2G152526 |
| Sobic.003G220700 | Putative uncharacterized protein (A0A1B6Q4P3) - Zinc finger protein 3-like | Shpool5c_g018 | No hit | Chr3A*, Chr3B* and +, Chr3D* and + | LOC_Os01g42970 | GRMZM2G039889 |
| Sobic.003G220600 | Putative uncharacterized protein (C5XR17) - Charged multivesicular body protein 4b | Shpool5c_g005 | No hit | Chr1A, Chr1B*,  Chr3A*, Chr3B* and +, Chr3D* and +, | LOC_Os09g09480 | GRMZM2G107757 |

**Supplementary Table 3.** Orthologous genes from Region02. Region02 orthologous genes found in the sugarcane BACs. “+” indicates that the chromosome has partial genes and “*” indicates more than one copy.

| Sorghum | Gene | BAC Genes | Sucest-Fun | S. Spontaneum [Zhang et al., 2018] | Rice | Maize |
| --- | --- | --- | --- | --- | --- | --- |
| Sobic.006G021400 | Putative uncharacterized protein (A0A1Z5RBR0) - Histidine kinase/Protein kinase (histidine) | Sh231B24_g0030 | No hit | Chr2A, Chr2B, Chr2C+, Chr2D,  Chr5A*, Chr5B*, Chr5C*, Chr5D* | LOC_Os04g13480 | GRMZM2G308046 |
| Sobic.004G229900 | Putative uncharacterized protein (Q651V4) - Ribosomal protein-like | Sh276O20_g0210, Sh431A16_g0040, Sh231B24_g0060 | SCQGST1031H03 | Chr1A, Chr1B, Chr1D,  Chr2A*, Chr2B, Chr2C*,  Chr3D,  Chr4A,  Chr5B*,  Chr6A*, Chr6B*, Chr6C,  Chr7B, Chr7D,  Chr8C* | LOC_Os01g40070 | GRMZM2G076892 |
| Sobic.004G230000 | Putative uncharacterized protein (A0A194YRF1) - Similar to NAM-like protein | Sh231B24_g0090, Sh276O20_g0160, Sh431A16_g0060 | SCQGLR1086C07 | Chr1A,  Chr2A*, Chr2B, Chr2C * and +,  Chr3D,  Chr4A+,  Chr5B*,  Chr6A, Chr6B * and +, Chr6C,  Chr7D,  Chr8C | LOC_Os06g02710 | GRMZM5G887243 |
| Sobic.008G134300 | Uncharacterized protein (C5YPX1) - Protein tyrosine kinase | Sh095J03_g0010 | SCJLST1024G01 | Chr2A* and +, Chr2B* and +, Chr2C* and +, Chr2D* and + | LOC_Os12g37980 | GRMZM2G332280 |
| Sobic.008G134401 | Uncharacterized protein (A0A1Z5R7M4) | Sh048L15_g0030, Sh431A16_g0090, Sh095J03_g0030, Sh255C13_g0020, Sh231B24_g0110, Sh276O20_g0150 | No hit | Chr2A+, Chr2C* | No hit | No hit |
| Sobic.008G134500 | Putative uncharacterized protein (A0A1Z5R6L9) - Protein-tyrosine kinase | Sh035E13_g0170, Sh095J03_g0210, Sh231B24_g0120, Sh255C13_g0040, Sh284G01_g0080, Sh431A16_g0140, Sh048L15_g0060, Sh218H04_g0010, Sh276O20_g0140 | SCSBAM1086F04 | Chr2A* and +, Chr2B* and +, Chr2C* and +, Chr2D* and +, | LOC_Os12g37980 | GRMZM2G332280 |
| Sobic.003G221600 | Putative uncharacterized protein (C5XR26) | Sh035E13_g0220, Sh040F02_g0030, Sh048L15_g0140, Sh095J03_g0310, Sh171E23_g0010, Sh218H04_g0060, Sh231B24_g0190, Sh255C13_g0080, Sh276O20_g0060, Sh284G01_g0110, Sh285K15_g0090, Sh431A16_g0220, Sh452C23_g0010 | SCCCSB1004A04 | Chr2D+,  Chr3B, Chr3C, Chr3D* and + | LOC_Os01g43060 | GRMZM2G056377 |
| Sobic.003G221500 | CENP-C2 (Q66LH1) - CENTROMERE PROTEIN C | Sh035E13_g0230, Sh040F02_g0060, Sh048L15_g0150, Sh095J03_g0330, Sh171E23_g0030, Sh218H04_g090, Sh255C13_g0120, Sh276O20_g0090, Sh284G01_g0120, Sh285K15_g0010, Sh431A16_g0250, Sh452C23_g0030 | SCSGFL4190C08 | Chr2A+, Chr2D+,  Chr3B, Chr3C, Chr3D* and +,  Chr7B+ | LOC_Os01g43050 | GRMZM2G114315 |
| Sobic.003G299500 | Uncharacterized protein (A0A1W0VZP0) | Sh035E13_g0240, Sh040F02_g0090, Sh048L15_g0170, Sh095J03_g0360, Sh171E23_g0060, Sh218H04_g0120, Sh255C13_g0130, Sh276O20_g0120, Sh284G01_g0130, Sh285K15_g0030, Sh431A16_g0280, Sh452C23_g0060 | SCJLAM1062D01 | Chr2A+, Chr2C* and +, Chr2D+,  Chr3A, Chr3C* and +, Chr3D* and + | LOC_Os01g55094 | GRMZM2G309660 |
| Sobic.008G134700 | Putative uncharacterized protein (C5YPX8) – Similar to aspartyl protease | Sh035E13_g0190, Sh048L15_g0130, Sh218H04_g0030, Sh231B24_g0160, Sh276O20_g0110, Sh284G01_g0090, Sh431A16_g0190, Sh255C13_g0380 | SCBFAD1048G09 | Chr2A* and +, Chr2C* and +, Chr2D* and + | LOC_Os05g31170 | GRMZM2G060680 |

**Supplementary Table 4.** Number of SNPs found in CENP-C and HP600. Summary of the sugarcane SNP counts by gene in the duplications in Region01 and Region02.

|  | | HP600 | | | CENPC | | | HP600 + CENPC | | |
| --- | --- | --- | --- | --- | --- | --- | --- | --- | --- | --- |
|  |  | Base length | SNPs | Bases/ SNP | Base length | SNPs | Bases/ SNP | Base length | SNPs | Bases/ SNP |
| Exonic Region | Region01 | 419 | 4 | 105 | 695 | 12 | 58 | 1114 | 16 | 70 |
|  | Region02 | 419 | 10 | 42 | 695 | 16 | 43 | 1114 | 26 | 43 |
|  | Region01 + Region02 | 419 | 24 | 17 | 695 | 38 | 18 | 1114 | 62 | 18 |
|  | Specific to Region | 419 | 10 | 42 | 695 | 10 | 70 | 1114 | 20 | 56 |
| Intronic Region | Region01 | 630 | 4 | 158 | 4703 | 131 | 36 | 5333 | 135 | 40 |
|  | Region02 | 630 | 17 | 37 | 4703 | 160 | 29 | 5333 | 177 | 30 |
|  | Region01 + Region02 | 630 | 36 | 18 | 4703 | 352 | 13 | 5333 | 388 | 14 |
|  | Specific to Region | 630 | 15 | 42 | 4703 | 61 | 77 | 5333 | 76 | 70 |
| Intronic + Exonic | Region01 | 1049 | 8 | 131 | 5398 | 143 | 38 | 6447 | 151 | 43 |
|  | Region02 | 1049 | 27 | 39 | 5398 | 176 | 31 | 6447 | 203 | 32 |
|  | Region01 + Region02 | 1049 | 60 | 17 | 5398 | 390 | 14 | 6447 | 450 | 14 |
|  | Specific to Region | 1049 | 25 | 42 | 5398 | 71 | 76 | 6447 | 96 | 67 |

**Supplementary Table 5.** Number of SNPs found in duplicated regions. Summary of the sugarcane SNP counts in duplications in Region01 and Region02.

|  | Intergenic | | | Whole duplication | | |
| --- | --- | --- | --- | --- | --- | --- |
|  | Base length | SNPs | Bases/ SNP | Base length | SNPs | Bases/ SNP |
| Region01 | 2513 | 80 | 31 | 8960 | 232 | 39 |
| Region02 | 2513 | 81 | 31 | 8960 | 284 | 32 |
| Region01 + Region02 | 2513 | 269 | 9 | 8960 | 719 | 12 |
| Specific per region | 2513 | 108 | 23 | 8960 | 203 | 44 |

**Supplementary Table 6.** Chromosome counts. Chromosome counts by sugarcane variety.

| Chromosomes | IACSP95-3018 | IACSP93-3046 | RB835486 | SP80-3280 | | SP81-3250 | |
| --- | --- | --- | --- | --- | --- | --- | --- |
| 98 | 0 | 0 | 0 | 0 | 6 | |  |
| 99 | 0 | 0 | 0 | 0 | 3 | |  |
| 100 | 0 | 0 | 3 | 0 | 6 | |  |
| 101 | 0 | 0 | 0 | 0 | 0 | |  |
| 102 | 2 | 0 | 5 | 0 | 6 | |  |
| 103 | 1 | 0 | 1 | 0 | 1 | |  |
| 104 | 7 | 0 | 0 | 0 | 1 | |  |
| 105 | 0 | 0 | 4 | 0 | 0 | |  |
| 106 | 5 | 2 | 5 | 0 | 5 | |  |
| 107 | 1 | 3 | 1 | 0 | 2 | |  |
| 108 | 8 | 3 | 8 | 2 | 3 | |  |
| 109 | 2 | 7 | 0 | 0 | 0 | |  |
| 110 | 9 | 11 | 7 | 2 | 6 | |  |
| 111 | 4 | 2 | 0 | 1 | 4 | |  |
| 112 | 11 | 13 | 15 | 5 | 7 | |  |
| 113 | 2 | 0 | 1 | 2 | 0 | |  |
| 114 | 1 | 3 | 3 | 1 | 2 | |  |
| 115 | 0 | 1 | 0 | 4 | 0 | |  |
| 116 | 0 | 1 | 3 | 2 | 0 | |  |
| 117 | 0 | 0 | 0 | 3 | 0 | |  |
| 118 | 0 | 0 | 0 | 2 | 0 | |  |
| Sum | 53 | 46 | 56 | 24 | 52 | |  |

**Supplementary Table 7.** Sequenom iPLEX MassARRAY® primers. SNPs derived from the HP600 and CENP-C duplicated regions genotyped in the population and the three primers used for genotyping on the SEQUENOM platform.

| SNP ID | Forward Primer ID | Forward Primer Sequence | Reverse Primer ID | Reverse Primer Sequence | Extended Primer ID | Extended Primer Sequence |
| --- | --- | --- | --- | --- | --- | --- |
| SugSNP_sh081 | SugSNP_Sh_081_W1_F | ACGTTGGATGGTCTGACAAAGATAATAAATG | SugSNP_Sh_081_W1_R | ACGTTGGATGCTTTTATTGGGCTTCTTTCC | SugSNP_Sh_081_W1_E | ACCCATCTCCGCGTCAT |
| SugSNP_sh099 | SugSNP_Sh_099_W1_F | ACGTTGGATGTCATTCAGAGCAAGCTGTGG | SugSNP_Sh_099_W1_R | ACGTTGGATGACTATCATCTTCCGAGTCAG | SugSNP_Sh_099_W1_E | TTCCGAGTCAGATGAGC |
| SugSNP_sh086 | SugSNP_Sh_086_W1_F | ACGTTGGATGTTCAATGGTGCAGTCAGCAG | SugSNP_Sh_086_W1_R | ACGTTGGATGGGGAGCTTGTTGGACATTTG | SugSNP_Sh_086_W1_E | AGTCAGCAGCTCTTCCTT |
| SugSNP_sh090 | SugSNP_Sh_090_W1_F | ACGTTGGATGATCTGCTCAAGTGTCGGTTC | SugSNP_Sh_090_W1_R | ACGTTGGATGCTAAGATCTTTTTCAGTGGC | SugSNP_Sh_090_W1_E | TTAAACCTTGTTTCCGTG |
| SugSNP_sh061 | SugSNP_Sh_061_W1_F | ACGTTGGATGCTTTACAGGAGCACCATGGG | SugSNP_Sh_061_W1_R | ACGTTGGATGGATGAAGGAGGCGGGAGGC | SugSNP_Sh_061_W1_E | GGAGCACCATGGGAGAGCC |
| SugSNP_sh015 | SugSNP_Sh_015_W1_F | ACGTTGGATGGCAGGCCATATTCTTGATCC | SugSNP_Sh_015_W1_R | ACGTTGGATGAACCAACTGAGGAACCTCTG | SugSNP_Sh_015_W1_E | TGATCCTGAACCATGCTTGC |
| SugSNP_sh043 | SugSNP_Sh_043_W1_F | ACGTTGGATGAGCACTTGAGCAGCAATGC | SugSNP_Sh_043_W1_R | ACGTTGGATGTTGCTGCTTCACCTATGCTC | SugSNP_Sh_043_W1_E | GTTTGGTTCACTTAGTGGTAC |
| SugSNP_sh037 | SugSNP_Sh_037_W1_F | ACGTTGGATGGGCCAAGATGGCAAGAGAAC | SugSNP_Sh_037_W1_R | ACGTTGGATGGGATTTAGCGACAAGATCTG | SugSNP_Sh_037_W1_E | AGAACATTGAAAGTGAAATCT |
| SugSNP_sh005 | SugSNP_Sh_005_W1_F | ACGTTGGATGAGCAAACCGATGCCTGTTG | SugSNP_Sh_005_W1_R | ACGTTGGATGAAAGTATTCAACTGGATCCG | SugSNP_Sh_005_W1_E | CCTGTTGTGGATCAATCTAAGTT |
| SugSNP_sh064 | SugSNP_Sh_064_W1_F | ACGTTGGATGACAAGGAGGGGAAGCGTAAG | SugSNP_Sh_064_W1_R | ACGTTGGATGAGAGCTAGTTCAACAGTACC | SugSNP_Sh_064_W1_E | GAGCTAGTTCAACAGTACCTTGGCT |
| SugSNP_sh066 | SugSNP_Sh_066_W2_F | ACGTTGGATGCAATTGCAGAACAAGCCTCC | SugSNP_Sh_066_W2_R | ACGTTGGATGTCTCTCTCACCATCTCAATG | SugSNP_Sh_066_W2_E | TACTCCTCGTTCCCAGT |
| SugSNP_sh035 | SugSNP_Sh_035_W2_F | ACGTTGGATGTGCCTGCAGTTATTGGCATC | SugSNP_Sh_035_W2_R | ACGTTGGATGTCTGAATACTGGTCAGGCAC | SugSNP_Sh_035_W2_E | TGGCATCAAAGCATACT |
| SugSNP_sh013 | SugSNP_Sh_013_W2_F | ACGTTGGATGACCAGATATTGTGATGGGTG | SugSNP_Sh_013_W2_R | ACGTTGGATGGGTCTCCCTGTCAATTTCAC | SugSNP_Sh_013_W2_E | TGTGATGGGTGAACCAT |
| SugSNP_sh016 | SugSNP_Sh_016_W2_F | ACGTTGGATGGCAGGCCATATTCTTGATCC | SugSNP_Sh_016_W2_R | ACGTTGGATGCACAAACCAACTGAGGAACC | SugSNP_Sh_016_W2_E | TGAGGAACCTCTGGATTG |
| SugSNP_sh083 | SugSNP_Sh_083_W2_F | ACGTTGGATGGATAATAAATGTAAAGGTCC | SugSNP_Sh_083_W2_R | ACGTTGGATGTCTGTCTTTTATTGGGCTTC | SugSNP_Sh_083_W2_E | TATTGGGCTTCTTTCCTTT |
| SugSNP_sh019 | SugSNP_Sh_019_W2_F | ACGTTGGATGGATGTGCCAATAGACTATCC | SugSNP_Sh_019_W2_R | ACGTTGGATGGCCCTCCAGATGATGAGAAG | SugSNP_Sh_019_W2_E | ACTATTGGCAGATCTACTAG |
| SugSNP_sh100 | SugSNP_Sh_100_W2_F | ACGTTGGATGTCTGACTCGGAAGATGATAG | SugSNP_Sh_100_W2_R | ACGTTGGATGTCTTTGTCAGACACGATAGG | SugSNP_Sh_100_W2_E | GGAAGATGATAGTGATGACA |
| SugSNP_sh088 | SugSNP_Sh_088_W2_F | ACGTTGGATGCTGCGATATCACATCTGCTC | SugSNP_Sh_088_W2_R | ACGTTGGATGCACGGAAACAAGGTTTAAAG | SugSNP_Sh_088_W2_E | TGCTCAAGTGTCGGTTCTCCTT |
| SugSNP_sh092 | SugSNP_Sh_092_W2_F | ACGTTGGATGGATCTTCATTCAGAGCAAGC | SugSNP_Sh_092_W2_R | ACGTTGGATGATCTTCCGAGTCAGATGAGC | SugSNP_Sh_092_W2_E | TTCATTCAGAGCAAGCTGTGGA |
| SugSNP_sh085 | SugSNP_Sh_085_W2_F | ACGTTGGATGAACGCTACATGCAACTCTGG | SugSNP_Sh_085_W2_R | ACGTTGGATGAGCTGCTGACTGCACCATTG | SugSNP_Sh_085_W2_E | CATTGAAAAAATTCTTTTGGTAAG |
| SugSNP_sh031 | SugSNP_Sh_031_W3_F | ACGTTGGATGATGAATCTAGCCATGCACTG | SugSNP_Sh_031_W3_R | ACGTTGGATGCATCATTATGAGGTTGATTC | SugSNP_Sh_031_W3_E | ACTGGAAATACCCCAAG |
| SugSNP_sh067 | SugSNP_Sh_067_W3_F | ACGTTGGATGGATCTTCATTCAGAGCAAGC | SugSNP_Sh_067_W3_R | ACGTTGGATGGGACCTTTACATTTATTATC | SugSNP_Sh_067_W3_E | TCAGAGCAAGCTGTGGA |
| SugSNP_sh004 | SugSNP_Sh_004_W3_F | ACGTTGGATGGGCTAAAATGGTGCTGAAGG | SugSNP_Sh_004_W3_R | ACGTTGGATGTGCTCTTTGCTGCCATTTGC | SugSNP_Sh_004_W3_E | GATGGAGTGAAAGCGAG |
| SugSNP_sh030 | SugSNP_Sh_030_W3_F | ACGTTGGATGCACCTGATTTGTGCAATG | SugSNP_Sh_030_W3_R | ACGTTGGATGTGCCCCTCTTTGACTGTTTC | SugSNP_Sh_030_W3_E | ACTGTTTCTTCATTTTCCC |
| SugSNP_sh042 | SugSNP_Sh_042_W3_F | ACGTTGGATGAGCACTTGAGCAGCAATGC | SugSNP_Sh_042_W3_R | ACGTTGGATGTTGCTGCTTCACCTATGCTC | SugSNP_Sh_042_W3_E | ACCACTAAGTGAACCAAAC |
| SugSNP_sh003 | SugSNP_Sh_003_W3_F | ACGTTGGATGACTCCTTCCGCGGACCCTT | SugSNP_Sh_003_W3_R | ACGTTGGATGACTGCAATGGCCTCGAGGA | SugSNP_Sh_003_W3_E | GCCTCGAGGAGCGCGTCGC |
| SugSNP_sh017 | SugSNP_Sh_017_W3_F | ACGTTGGATGGAGGTTCCTCAGTTGGTTTG | SugSNP_Sh_017_W3_R | ACGTTGGATGCACAGAAATATTGCTCCTCC | SugSNP_Sh_017_W3_E | GTTTGTGCAGAGACACAGA |
| SugSNP_sh052 | SugSNP_Sh_052_W3_F | ACGTTGGATGCTGACATAATCACATAACAC | SugSNP_Sh_052_W3_R | ACGTTGGATGGGCAAAGCTGGAAAAGATAC | SugSNP_Sh_052_W3_E | AACACTATTCAAAGTTCTGTT |
| SugSNP_sh012 | SugSNP_Sh_012_W3_F | ACGTTGGATGGCCAGGATCGAATTTCAGAG | SugSNP_Sh_012_W3_R | ACGTTGGATGTCTGGTGAACCTTCAGATTC | SugSNP_Sh_012_W3_E | TAGAGGTGCTGCAAATGCTAA |
| SugSNP_sh091 | SugSNP_Sh_091_W3_F | ACGTTGGATGCTGATTTGTGATACAGGGAG | SugSNP_Sh_091_W3_R | ACGTTGGATGTGCTGATGCAAGACAACCTG | SugSNP_Sh_091_W3_E | TACAGGGAGGACCGTGAAAACA |
| SugSNP_sh001 | SugSNP_Sh_001_W4_F | ACGTTGGATGACTCCTTCCGCGGACCCTT | SugSNP_Sh_001_W4_R | ACGTTGGATGACTGCAATGGCCTCGAGGA | SugSNP_Sh_001_W4_E | GACCCTTGGCCCCGCCC |
| SugSNP_sh084 | SugSNP_Sh_084_W4_F | ACGTTGGATGCTTTACAGGAGCACCATGGG | SugSNP_Sh_084_W4_R | ACGTTGGATGCAAGCCTACCGATGAAGGAG | SugSNP_Sh_084_W4_E | AGCACCATGGGAGAGCC |
| SugSNP_sh036 | SugSNP_Sh_036_W4_F | ACGTTGGATGTGCCTGCAGTTATTGGCATC | SugSNP_Sh_036_W4_R | ACGTTGGATGTCTGAATACTGGTCAGGCAC | SugSNP_Sh_036_W4_E | TCTCTTGCCATCTTGGCC |
| SugSNP_sh087 | SugSNP_Sh_087_W4_F | ACGTTGGATGTTCAATGGTGCAGTCAGCAG | SugSNP_Sh_087_W4_R | ACGTTGGATGGGGAGCTTGTTGGACATTTG | SugSNP_Sh_087_W4_E | TGTTGGTGAGTTTTGGAA |
| SugSNP_sh065 | SugSNP_Sh_065_W4_F | ACGTTGGATGGGTATTCTTTCGTGTGCCAG | SugSNP_Sh_065_W4_R | ACGTTGGATGCAATCTCCCCCAAGAAATCC | SugSNP_Sh_065_W4_E | AAGAAATCCTTGACTTTCC |
| SugSNP_sh082 | SugSNP_Sh_082_W4_F | ACGTTGGATGGATAATAAATGTAAAGGTCC | SugSNP_Sh_082_W4_R | ACGTTGGATGTCTGTCTTTTATTGGGCTTC | SugSNP_Sh_082_W4_E | ATGACGCGGAGATGGGTCC |
| SugSNP_sh014 | SugSNP_Sh_014_W4_F | ACGTTGGATGACCAGATATTGTGATGGGTG | SugSNP_Sh_014_W4_R | ACGTTGGATGGGTCTCCCTGTCAATTTCAC | SugSNP_Sh_014_W4_E | GCATGATTCTTCTGATGTTC |
| SugSNP_sh018 | SugSNP_Sh_018_W4_F | ACGTTGGATGGAGGTTCCTCAGTTGGTTTG | SugSNP_Sh_018_W4_R | ACGTTGGATGCACAGAAATATTGCTCCTCC | SugSNP_Sh_018_W4_E | TGCATGCCTCGTTTTCTTTGG |
| SugSNP_sh102 | SugSNP_Sh_102_W4_F | ACGTTGGATGTCTGACTCGGAAGATGATAG | SugSNP_Sh_102_W4_R | ACGTTGGATGTCTTTGTCAGACACGATAGG | SugSNP_Sh_102_W4_E | TGTCAGACACGATAGGTTTGTC |
| SugSNP_sh006 | SugSNP_Sh_006_W4_F | ACGTTGGATGAGCAAACCGATGCCTGTTG | SugSNP_Sh_006_W4_R | ACGTTGGATGAAAGTATTCAACTGGATCCG | SugSNP_Sh_006_W4_E | ATTCAACTGGATCCGAAATATTC |
| SugSNP_sh063 | SugSNP_Sh_063_W5_F | ACGTTGGATGACAAGGAGGGGAAGCGTAAG | SugSNP_Sh_063_W5_R | ACGTTGGATGAGAGCTAGTTCAACAGTACC | SugSNP_Sh_063_W5_E | AGCGTAAGTCGGGGCCG |
| SugSNP_sh089 | SugSNP_Sh_089_W5_F | ACGTTGGATGCTGCGATATCACATCTGCTC | SugSNP_Sh_089_W5_R | ACGTTGGATGCACGGAAACAAGGTTTAAAG | SugSNP_Sh_089_W5_E | ACAAGGTTTAAAGTATTGGG |
| SugSNP_sh011 | SugSNP_Sh_011_W5_F | ACGTTGGATGTTCATTAGCTGAGAAGGATG | SugSNP_Sh_011_W5_R | ACGTTGGATGCTTCCTCTTCAGATTCATCC | SugSNP_Sh_011_W5_E | AGGTCAGTAAATAAGTCAAATC |
| SugSNP_sh080 | SugSNP_Sh_080_W5_F | ACGTTGGATGTCATTCAGAGCAAGCTGTGG | SugSNP_Sh_080_W5_R | ACGTTGGATGGGACCTTTACATTTATTATC | SugSNP_Sh_080_W5_E | ACATTTATTATCTTTGTCAGACA |

# References

Cardoso-Silva, C.B., Costa, E.A., Mancini, M.C., Balsalobre, T.W., Canesin, L.E., Pinto, L.R., et al. (2014). De novo assembly and transcriptome analysis of contrasting sugarcane varieties. PLoS One 9, e88462.

Chalhoub, B., Belcram, H., and Caboche, M. (2004). Efficient cloning of plant genomes into bacterial artificial chromosome (BAC) libraries with larger and more uniform insert size. Plant Biotechnol. J. 2, 181-188.

de Setta, N., Monteiro-Vitorello, C.B., Metcalfe, C.J., Cruz, G.M., Del Bem, L.E., Vicentini, R., et al. (2014). Building the sugarcane genome for biotechnology and identifying evolutionary trends. BMC Genomics 15, 540.

Ewing, B., and Green, P. (1998). Base-calling of automated sequencer traces using phred. II. Error probabilities. Genome Res. 8, 186-194.

Ewing, B., Hillier, L., Wendl, M.C., and Green, P. (1998). Base-calling of automated sequencer traces using phred. I. Accuracy assessment. Genome Res. 8, 175-185.

Felsenstein, J. (1985). Confidence limits on phylogenies: an approach using the bootstrap. Evolution 39, 783-791.

Figueira, T.R., Okura, V., da Silva, F.R., da Silva, M.J., Kudrna, D., Ammiraju, J.S., et al. (2012). A BAC library of the SP80-3280 sugarcane variety (saccharum sp.) and its inferred microsynteny with the sorghum genome. BMC Res. Notes 5, 185.

Garsmeur, O., Charron, C., Bocs, S., Jouffe, V., Samain, S., Couloux, A., et al. (2011). High homologous gene conservation despite extreme autopolyploid redundancy in sugarcane. New Phytol. 189, 629-642.

Gonthier, L., Bellec, A., Blassiau, C., Prat, E., Helmstetter, N., Rambaud, C., et al. (2010). Construction and characterization of two BAC libraries representing a deep-coverage of the genome of chicory (Cichorium intybus L., Asteraceae). BMC Res. Notes 3, 225.

Goodstein, D.M., Shu, S., Howson, R., Neupane, R., Hayes, R.D., Fazo, J., et al. (2012). Phytozome: a comparative platform for green plant genomics. Nucleic Acids Res. 40, D1178-D1186.

Gordon, D. (2003). "Viewing and editing assembled sequences using consed," in Current Protocols in Bioinformatics, eds. D. Baxevanis and D. Davison (New York, NY: John Wiley), 1-43.

Gordon, D., Abajian, C., and Green, P. (1998). Consed: a graphical tool for sequence finishing. Genome Res. 8, 195-202.

Gordon, D., Desmarais, C., and Green, P. (2001). Automated finishing with autofinish. Genome Res. 11, 614-625.

Guindon, S., and Gascuel, O. (2003). A simple, fast, and accurate algorithm to estimate large phylogenies by maximum likelihood. Syst. Biol. 52, 696-704.

Kim, C., Lee, T.H., Compton, R.O., Robertson, J.S., Pierce, G.J., and Paterson, A.H. (2013). A genome-wide BAC end-sequence survey of sugarcane elucidates genome composition, and identifies BACs covering much of the euchromatin. Plant Mol. Biol. 81, 139-147.

Jannoo, N., Grivet, L., Chantret, N., Garsmeur, O., Glaszmann, J.C., Arruda, P., et al. (2007). Orthologous comparison in a gene-rich region among grasses reveals stability in the sugarcane polyploid genome. Plant J. 50, 574-585.

Katoh, K., Misawa, K., Kuma, K., and Miyata, T. (2002). MAFFT: a novel method for rapid multiple sequence alignment based on fast Fourier transform. Nucleic Acids Res. 30, 3059-3066.

Kimura, M. (1980). A simple method for estimating evolutionary rates of base substitutions through comparative studies of nucleotide sequences. J. Mol. Evol. 16, 111-120.

Kriventseva, E. V., Kuznetsov, D., Tegenfeldt, F., Manni, M., Dias, R., Simão, F. A., & Zdobnov, E. M. (2018). OrthoDB v10: sampling the diversity of animal, plant, fungal, protist, bacterial and viral genomes for evolutionary and functional annotations of orthologs. Nucleic acids research, 47(D1), D807-D811.

Kumar, S., Stecher, G., and Tamura, K. (2016). MEGA7: molecular evolutionary genetics analysis version 7.0 for bigger datasets. Mol. Biol. Evol. 33, 1870-1874.

Mancini, M.C., Cardoso-Silva, C.B., Sforca, D.A., and de Souza, A.P. (2018). "Targeted sequencing by gene synteny," a new strategy for polyploid species: sequencing and physical structure of a complex sugarcane region. Front. Plant Sci. 9, 397.

Murray, S.C., Sharma, A., Rooney, W.L., Klein, P.E., Mullet, J.E., Mitchell, S.E., et al. (2008). Genetic improvement of sorghum as a biofuel feedstock: I. QTL for stem sugar and grain nonstructural carbohydrates. Crop Science 48, 2165-2179.

Paiva, J.A., Prat, E., Vautrin, S., Santos, M.D., San-Clemente, H., Brommonschenkel, S., et al. (2011). Advancing Eucalyptus genomics: identification and sequencing of lignin biosynthesis genes from deep-coverage BAC libraries. BMC Genomics 12, 137.

Paux, E., Sourdille, P., Salse, J., Saintenac, C., Choulet, F., Leroy, P., et al. (2008). A physical map of the 1-gigabase bread wheat chromosome 3B. Science 322, 101-104.

Peterson, D.G., Tomkins, J.P., Frisch, D.A., Wing, R.A., and Paterson, A. (2000). Construction of plant bacterial artificial chromosome (BAC) libraries: an illustrated guide. J. Agric. Genomics 5, 1-100.

Riaño-Pachón, D.M., and Mattiello, L. (2017). Draft genome sequencing of the sugarcane hybrid SP80-3280. F1000Research 6, 861.

Roselli, S., Olry, A., Vautrin, S., Coriton, O., Ritchie, D., Galati, G., et al. (2017). A bacterial artificial chromosome (BAC) genomic approach reveals partial clustering of the furanocoumarin pathway genes in parsnip. Plant J. 89, 1119-1132.

Saitou, N., and Nei, M. (1987). The neighbor-joining method: a new method for reconstructing phylogenetic trees. Mol. Biol. Evol. 4, 406-425.

Talbert, P.B., Bryson, T.D., and Henikoff, S. (2004). Adaptive evolution of centromere proteins in plants and animals. J. Biol. 3, 18.

Vettore, A.L., da Silva, F.R., Kemper, E.L., Souza, G.M., da Silva, A.M., Ferro, M.I., et al. (2003). Analysis and functional annotation of an expressed sequence tag collection for tropical crop sugarcane. Genome Res. 13, 2725-2735.

Vilela, M.M., Del Bem, L.E., Van Sluys, M.A., de Setta, N., Kitajima, J.P., Cruz, G.M., et al. (2017). Analysis of three sugarcane homo/homeologous regions suggests independent polyploidization events of *Saccharum officinarum* and *Saccharum spontaneum*. Genome Biol. Evol. 9, 266-278.

Visendi, P., Berkman, P.J., Hayashi, S., Golicz, A.A., Bayer, P.E., Ruperao, P., et al. (2016). An efficient approach to BAC based assembly of complex genomes. Plant Methods 12, 2.

Wang, J., Roe, B., Macmil, S., Yu, Q., Murray, J.E., Tang, H., et al. (2010). Microcollinearity between autopolyploid sugarcane and diploid *Sorghum* genomes. BMC Genomics 11, 261.

Zhang, J., Zhang, X., Tang, H., Zhang, Q., Hua, X., Ma, X., et al. (2018b). Publisher correction: allele-defined genome of the autopolyploid sugarcane *Saccharum spontaneum L*. Nat. Genet. 50, 1754.
